# Zero‐Shot Self‐Supervised Learning of Single Breath‐Hold Magnetic Resonance Cholangiopancreatography (MRCP) Reconstruction

**DOI:** 10.1002/mrm.70467

**Published:** 2026-06-12

**Authors:** Jinho Kim, Marcel Dominik Nickel, Florian Knoll

**Affiliations:** ^1^ Department Artificial Intelligence in Biomedical Engineering Friedrich‐Alexander‐Universität Erlangen‐Nürnberg Erlangen Germany; ^2^ Research and Clinical Translation, Magnetic Resonance, Siemens Healthineers AG Erlangen Germany

**Keywords:** breath‐hold MRCP, deep learning–based MRI reconstruction, MR cholangiopancreatography, self‐supervised training, zero‐shot learning

## Abstract

**Purpose:**

To investigate the feasibility of zero‐shot self‐supervised learning reconstruction for reducing breath‐hold times in magnetic resonance cholangiopancreatography (MRCP).

**Methods:**

Breath‐hold MRCP was acquired from 11 healthy volunteers on 3T scanners using an incoherent *k*‐space sampling pattern, leading to a 14‐s acquisition time and an acceleration factor of *R* = 25. Zero‐shot reconstruction was compared with parallel imaging of respiratory‐triggered MRCP (338 s, *R* = 3) and compressed sensing reconstruction. For two volunteers, breath‐hold scans (40 s, *R* = 6) were additionally acquired and retrospectively undersampled to *R* = 25 to compute peak signal‐to‐noise ratio (PSNR). To address long zero‐shot training time, the n+m full stages of the zero‐shot learning were divided into two parts to reduce backpropagation depth during training: (1) n frozen stages initialized with n‐stage pretrained network and (2) m trainable stages initialized either randomly or m‐stage pretrained network. Efficiency of our approach was assessed by varying initialization strategies and the number of trainable stages using the retrospectively undersampled data.

**Results:**

Zero‐shot reconstruction significantly improved visual image quality over compressed sensing, particularly in SNR and ductal delineation, and achieved image quality comparable to that of successful respiratory‐triggered acquisitions with regular breathing patterns. Improved initializations enhanced PSNR and reduced reconstruction time. Adjusting frozen/trainable configurations demonstrated that PSNR decreased only slightly from 38.25 dB (0/13) to 37.67 dB (12/1), while training time decreased up to 6.7‐fold.

**Conclusion:**

Zero‐shot learning delivers high‐fidelity MRCP reconstructions with reduced breath‐hold times, and the proposed partially trainable approach offers a practical solution for translation into time‐constrained clinical workflows.

## 1 Introduction

Magnetic resonance cholangiopancreatography (MRCP) is a noninvasive imaging technique used to visualize the biliary and pancreatic ductal systems, playing a critical role in the diagnosis of hepatobiliary diseases [1, 2, 3, 4]. Traditionally performed using 2D thick‐slab acquisitions, MRCP has evolved toward high‐resolution 3D acquisitions to provide comprehensive anatomical detail [5]. However, this transition has come at the cost of significantly longer acquisition times, which increase susceptibility to motion artifacts and reduce image quality, particularly in free‐breathing with uncooperative patients [6, 7, 8].

To mitigate these effects, respiratory‐triggered acquisitions in free‐breathing have been widely adopted. Techniques like prospective acquisition correction (PACE) [9] allow for improved motion suppression by synchronizing data acquisition with the patient's respiratory cycle, offering clearer anatomical contours and better patient tolerance. However, respiratory‐triggered MRCP still suffers from prolonged and unpredictable scan durations, especially in patients with irregular or shallow breathing patterns [8, 10, 11, 12], resulting in blurry images.

Breath‐hold MRCP offers an alternative approach by acquiring data during a short breath‐hold period, thereby eliminating respiratory motion artifacts [13, 14, 15, 16, 17, 18, 19, 20]. $\ell_{1}$‐wavelet compressed sensing (CS) reconstruction [21] was proposed for breath‐hold MRCP due to the intrinsic sparsity of biliary structures [17, 18, 19, 20]. However, it has been reported that it often fails to depict ductal details [19], which limits diagnostic confidence in assessing strictures or dilations. It also requires breath‐hold durations of approximately 20 s [10, 14, 15], which may be challenging for patients that have limited breath‐hold capability [22]. To address these limitations, various strategies have been proposed, such as modifying the CS protocol by reducing the field‐of‐view [19] and training patients in breath‐holding techniques before scanning [20, 22, 23]. However, reducing the field‐of‐view may fail to adequately cover the region of interest, and training patients before every scan is not trivial. Therefore, optimizing the *k*‐space sampling patterns [24, 25], such as a combination of equidistance and incoherent random undersampling offers a more practical and effective solution.

Deep learning–based MRI reconstructions have shown convincing results in accelerated MRI, particularly through physics‐driven unrolled networks that integrate data fidelity and learned regularization [26, 27, 28, 29]. These models typically require a large number of fully sampled training examples [26, 27], which are difficult to obtain for applications in the abdomen like MRCP, where longer acquisitions are increasingly likely to be corrupted by motion artifacts. Yaman et al. proposed self‐supervised deep learning reconstruction methods to eliminate the need for a fully sampled ground truth (self‐supervised learning via data undersampled [SSDU]^28^) and even to remove the need for training data at all by training a model for a single specific acquisition (zero‐shot self‐supervised learning [29]).

Several scan‐specific reconstruction approaches that learn directly from the acquired data without large external databases have been proposed. The zero‐shot self‐supervised approach shares the data‐specific nature of these methods but differs fundamentally by combining physics‐guided unrolled optimization with self‐supervised loss partitioning. Unlike RAKI [30] or Residual‐RAKI [31], it operates in the image domain with explicit data consistency terms rather than direct *k*‐space interpolation. In contrast to deep image prior [32] (DIP) or implicit neural representations [33, 34] (INR), it does not rely on the implicit bias of network architecture alone but enforces data consistency with measured data through the MRI encoding operator. Thus, our work bridges model‐based deep learning and scan‐specific adaptation, demonstrating zero‐shot self‐supervised reconstruction for highly accelerated 3D breath‐hold MRCP.

In this work, we investigate the technical feasibility of highly accelerated breath‐hold MRCP reconstruction using zero‐shot self‐supervised learning. By combining a short 14‐s breath‐hold acquisition with scan‐specific reconstruction, we demonstrate improved image quality relative to conventional CS without relying on external training datasets. One critical downside of zero‐shot learning is the long training time of several hours per scan [29, 35], which hinders its clinical application. We developed a training strategy that leverages a pretrained reconstruction backbone by freezing the early stages of a self‐supervised network and updating only the last trainable stages during zero‐shot training. This reduces backpropagation depth and computation time with only a minimal trade‐off in image quality.

## 2 Methods

### 2.1 Zero‐Shot Self‐Supervised Learning for MRCP Reconstruction

Zero‐shot reconstruction is designed for subject‐specific self‐supervised learning using only a single dataset [29]. In zero‐shot learning, the acquired sampling pattern Ω is subdivided into three subsets of Tfor training,Λfor loss,andΓfor self−validation, and the available measurement samples from a single dataset are partitioned as:

(1) Ω=T⊔Λ⊔Γ,

where ⊔ denotes a disjoint union, that is, T,Λ, and Γ are mutually exclusive to each other.

Each volumetric acquisition can be decoupled in readout direction using a Fourier transformation. The resulting D readout positions, indexed by j, correspond to 2D *k*‐space datasets in the phase encoding plane. This approach allows for more training samples and reduces memory requirements, assuming a neural network–based regularization that correlates pixels only in the phase encode directions.

For each readout index j∈{1,…,D}, multiple disjoint mask pairs $\left(T_{k}^{j},\Lambda_{k}^{j}\right)$ are generated within $\Omega^{j}\setminus \Gamma^{j}$, where k∈{1,…,K}. These define the training dataset for zero‐shot learning as:

(2) $$\left(y_{T}^{\mathrm{jk}},y_{\Lambda }^{\mathrm{jk}},y_{\Gamma }^{j}\right):j\in \left\{1,\ldots ,D\right\},k\in \left\{1,\ldots ,K\right\},$$

where y is multi‐coil undersampled *k*‐space data. Zero‐shot reconstruction then minimizes the loss function

(3) $$\underset{\theta ,\lambda }{\text{argmin}}\frac{1}{\mathrm{DK}}\sum_{j=1}^{D}\sum_{k=1}^{K}ℒ\left(y_{\Lambda }^{\mathrm{jk}},A_{\Lambda }^{\mathrm{jk}}\left(f\left(y_{T}^{\mathrm{jk}},A_{T}^{\mathrm{jk}};\theta ,\lambda \right)\right)\right),$$

where f(·) is the output reconstruction image of the unrolled network parametrized with θ, and A the encoding operator that contains undersampling, Fourier transform, and coil sensitivities. The regularization parameter λ is learned during training [26], and ℒ(·) denotes a $\ell_{1}-\ell_{2}$ loss function [28].

**FIGURE 1:**
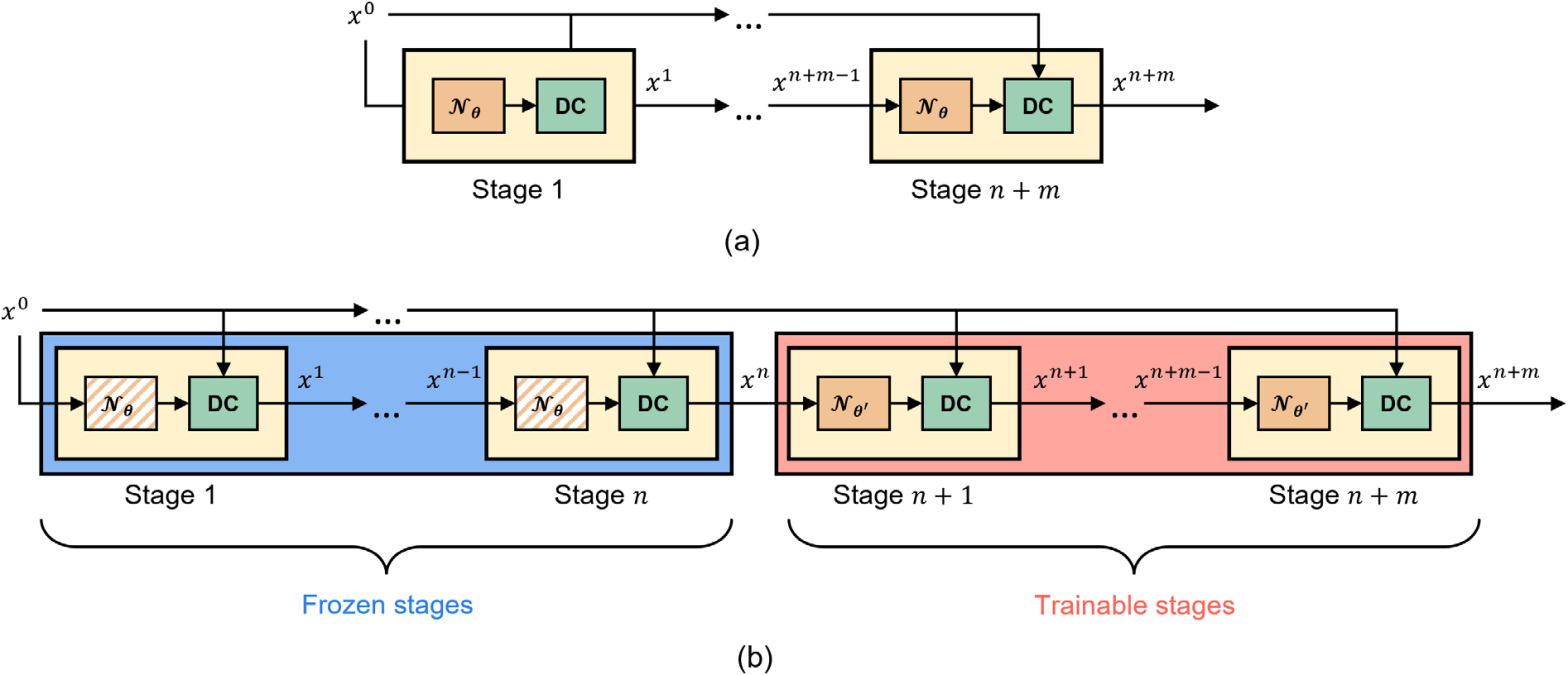
(a) Unrolled neural network architecture with n+m iterative stages. Each stage includes a regularization term (𝒩) and conjugate gradient‐based data consistency (DC) block. (b) Partially trainable architecture using a pretrained reconstruction model: The initial n stages are frozen (indicated by a patterned background), and later m stages with a new neural network (${𝒩}_{\theta^{\prime }}$) are trainable.

The long reconstruction times of zero‐shot learning arise because deep neural network models must be trained individually for each subject, often requiring extensive optimization over many unrolled stages. Although transfer learning can accelerate convergence compared to random‐initialized models [29], it still requires backpropagation through the entire unrolled architecture and repeated conjugate gradient‐based data consistency computations at every stage (Figure 1a), which contributes heavily to memory and runtime costs.

To address this, we developed an alternative training approach that split a total n+m stages of the unrolled network into n frozen stages and m trainable stages, where each stage was composed of a regularization block (𝒩) and a data consistency block (Figure 1b). The network ${𝒩}_{\theta }$ for the initial n frozen stages were initialized with an n‐stage pretrained network and their weights were frozen during zero‐shot training. The weights for the m trainable stages of the network ${𝒩}_{\theta^{\prime }}$ were initialized either randomly or with an m‐stage pretrained network and updated during training. We used SSDU^28^ to obtain the pretrained backbone in this study, but in principle any training strategy can be applied. This design eliminates the need for backpropagation through the entire unrolled network. Since the frozen network deterministically maps the undersampled input ($x^{0}$) to an intermediate reconstruction ($x^{n}$), $x^{n}$ was cached during the first epoch to avoid redundant computation in the frozen part. Training then takes the form,

(4) $$\underset{\theta ,\lambda }{\text{argmin}}\frac{1}{\mathrm{DK}}\sum_{j=1}^{D}\sum_{k=1}^{K}ℒ\left(y_{\Lambda }^{\mathrm{jk}},A_{\Lambda }^{\mathrm{jk}}\left(f^{\prime }\left({y^{\prime }}_{T}^{\mathrm{jk}},A_{T}^{\mathrm{jk}};\theta^{\prime },\lambda^{\prime }\right)\right)\right),$$

where $y^{\prime }$ denotes the precomputed input *k*‐space of $x^{n}$ in Figure 1b, $f^{\prime }$ indicates the model in the trainable stages, and $\lambda^{\prime }$ is the trainable regularization parameter. In this setting, only the trainable stages are involved in gradient computation during training, significantly reducing backpropagation depth, memory consumption, and total training time.

### 2.2 Data

All participants in this study were informed about the study objectives and data handling procedures and subsequently provided written consent for participation and further data proc essing. There were two independent datasets used to train the pretrained model (SSDU) and the zero‐shot learning, respectively.

#### 2.2.1 Dataset for the Pretrained Model

The pretrained model was obtained using the subset of 3 T acquisition from the raw dataset introduced in our previous study [27]. This dataset was collected from 31 healthy volunteers (19 males and 12 females) using 3T MRI scanners (MAGNETOM Vida and MAGNETOM Lumina, Siemens Healthineers AG, Forchheim, Germany) with a parallel imaging acceleration of *R* = 2 and PACE triggering.

#### 2.2.2 Dataset for Zero‐Shot Learning

We acquired MRCP data from 11 healthy volunteers (nine males and two females) between June and November 2025 on 3T MRI scanners (MAGNETOM Lumina and MAGNETOM Prisma, Siemens Healthineers AG, Forchheim, Germany) using 12‐channel body and 24‐channel spine receive array coils. The age distribution of the volunteers ranged from 27 to 83 years, with a mean age of 43.5±19.8 years. A 3D T2‐weighted turbo spin‐echo sequence (3D SPACE) [36] was used for both respiratory‐triggered and breath‐hold MRCP. Detailed protocol parameters are given in Table 1.

**TABLE 1:** Protocol parameters for breath‐hold MRCP and respiratory‐triggered MRCP.

	Breath‐hold	Respiratory‐triggered
Sequence	3D T2‐weighted TSE (3D SPACE)	
Acquisition plane	Coronal	
TR (ms)	2000	3165−6852 ^a^
TE (ms)	697	703
TA (s)	14	209−452 ^a^
Acquired voxel size ($mm^{3}$)	0.5×0.5×1.0	
Number of slices	64	
Flip angles (°)	100	105−120 ^a^
Number of signal averages	1.0	1.5
Triggering	N/A	PACE
Number of ACS lines	24	
Total acceleration factor	25	3

*Note:* The notation format for TR, TA, and Flip angles is Minimum−Maximum.

Abbreviations: ACS, autocalibration signal; N/A, not applicable; PACE, prospective acquisition correction; TA, acquisition time; TE, echo time; TR, repetition time; TSE, turbo spin echo.

^a^ Variable depending on the volunteer.

Respiratory‐triggered MRCP was acquired using a vendor‐provided sequence with PACE triggering and parallel imaging acceleration with an undersampling factor of *R* = 3 and 24 autocalibration lines. In this study, the goal of the respiratory‐triggered acquisitions was to serve as an image quality reference against which we compared our approach. To obtain the best possible image quality, we obtained multiple acquisitions in the same volunteer until we were able to obtain an acquisition with a sufficiently regular breathing pattern. This approach resulted in two scans for volunteer #08, three scans for volunteers #07 and #10, and four scans for volunteer #11. A single scan was sufficient for remaining volunteers.

For breath‐hold MRCP, we used a modified CS sequence [24, 25], combining 2D Poisson‐disk incoherent undersampling with equidistance undersampling and partial Fourier acquisition. Specifically, the 2D Poisson‐disk sampling pattern included additional equidistant undersampling factors of *R* = 3 and *R* = 2 for phase‐ and partition‐encoding directions, respectively. Partial Fourier acquisition was then applied, retaining 64% of *k*‐space in the in‐plane phase‐encoding direction and 67% in the partition‐encoding direction. This resulted in a total undersampling factor of *R* = 25 and a single 14 s breath‐hold acquisition. The undersampling pattern for breath‐hold MRCP is provided in Figure S1. Reference scan was conducted separately with center 24 autocalibration lines on the phase encoding plane. For quantitative analysis, we additionally acquired 40‐s breath‐hold scans at *R* = 6 from two of 11 volunteers in our study using the same sampling strategy and retrospectively undersampled to *R* = 25.

### 2.3 Data Preparation

Raw data was extracted and converted into the ISMRMRD format [37] using the pyMapVBVD Python package [38]. Due to the memory constraint for 3D reconstructions, we decoupled the fully sampled readout direction from the two phase‐encoding directions by applying an inverse Fourier transform, followed by volume‐wise normalization of the resulting stack of 2D *k*‐space data using the maximum absolute value of all samples in the stack.

Coil sensitivity maps were pre‐calculated using the ESPIRiT algorithm [39] using SigPy [40], with 24×24 fully sampled central *k*‐space data from the reference scan, a 5×5 kernel, and no background cropping in the image domain. The same coil sensitivity maps were consistently used across all model trainings and reconstructions to maintain consistency.

### 2.4 Deep Learning Reconstructions

All training was conducted on a Linux system equipped with NVIDIA A100 40GB GPU devices. The source code for zero‐shot learning reconstructions is available at https://github.com/JinhoKim46/ZS_BH‐MRCP.

#### 2.4.1 Unrolled Network

All deep learning reconstruction models in this study were based on unrolling an iterative algorithm. Each stage consisted of a learnable regularization module 𝒩, and a conjugate gradient‐based data consistency block (Figure 1a). 𝒩 followed a ResNet [41] architecture, consisting of eight residual blocks with 64 channels, resulting in approximately 628 k trainable parameters. The weights of 𝒩 were shared across all stages. All models were optimized using a normalized $\ell_{1}-\ell_{2}$ loss in the *k*‐space domain and trained with a cosine annealing learning rate schedule [42], starting from 0.0003. Notably, the learning rate was optimized to 0.0001 for volunteer #11 to ensure adequate image quality.

#### 2.4.2 Pretrained Model

By acquiring multiple scans from some volunteers, the training dataset consisted of 39 data volumes for training, 4 volumes for validation, and 18 volumes for testing. 1D Gaussian sampling strategy was applied to split the acquired sampling set Ω into T and Λ using a ratio $\rho_{\Lambda }=0.4$ [28]. We performed retrospective undersampling to *R* = 25 using the same breath‐hold MRCP sampling pattern shown in Figure S1. The number of unrolled stages of pretrained models was adjusted to match their target networks.

#### 2.4.3 Zero‐Shot Learning

To train the zero‐shot model, we followed the masking strategy of Yaman et al. [29], partitioning the acquired breath‐hold MRCP *k*‐space data Ω into three mutually exclusive subsets: Γ, Λ, and T, with respective splitting ratios $\rho_{\Gamma }=0.2$ and $\rho_{\Lambda }=0.4$. The remaining data were assigned to T. For each readout index j, K=10 independent $\left(T_{k}^{j},\Lambda_{k}^{j}\right)$ pairs were generated from $\Omega^{j}\setminus \Gamma^{j}$. Model training used early stopping criteria with a patience of three epochs and a minimum delta of $5\times 10^{-3}$ based on the validation loss computed on Γ to prevent overfitting during plateau phases. The regularization parameter λ in Equation (3) was learned along with the model parameters θ to control the balance between the data consistency term and the learned prior within the unrolled network during training.

#### 2.4.4 Numerical Experiments

We have chosen a total number of unrolled stages n+m of 13 for our experiment. We compared zero‐shot reconstructions of which trainable stages were initialized with either random weights or weights from an m‐stage pretrained network to evaluate the effect of parameter initialization. We further investigated the effect of initialization for the initial image as zero‐filled Fourier transform reconstruction, $\ell_{1}$‐wavelet CS reconstruction or generated by an n‐stage pretrained network. In this setup, $x^{n}$ in Figure 1b represented the output produced by the selected initialization method.

To quantify the quality‐computation trade‐off, we evaluated lightweight zero‐shot strategies on two retrospectively undersampled breath‐hold MRCP datasets (*R* = 25). Two experimental settings were considered. First, the total number of stages was fixed to 13, while varying the number of n frozen and m trainable stages, resulting in configurations ranging from 0/13 to 12/1. Second, the number of frozen stages was fixed to zero (n=0) and the number of m trainable stages was varied to analyze the effect of network depth on convergence behavior and reconstruction time.

An asterisk (*) was used as a label to indicate that the network for the trainable stages was initialized with the pretrained weights.

#### 2.4.5 Deep Learning‐Based Scan‐Specific Reconstructions

We compared zero‐shot reconstructions with other deep learning‐based scan‐specific reconstructions, such as DIP^32^ and hash‐based INR [34] reconstructions. Since both DIP and INR reconstructions do not have a stopping criterion, a fixed number of iterations per sample was heuristically determined. In addition, hyperparameters for each model were defined empirically to provide optimal image impression. Implementation details for both reconstructions are provided in Supporting Information Section B. The source code for DIP and INR implementations is also publicly available in our Git repository.

### 2.5 Conventional Reconstructions

For reference, we reconstructed our breath‐hold MRCP acquisitions with $\ell_{1}$‐wavelet CS using the SigPy [40]. The regularization parameter was set to 0.008 based on empirical visual assessment, selecting the value that provided the most appropriate balance between data fidelity and noise suppression while preserving natural visualization of ductal structures without over‐regularization. To reconstruct the respiratory‐triggered acquisitions, we used GRAPPA [43] using pygrappa [44].

### 2.6 Evaluation

To evaluate reconstruction performance, we qualitatively compared our proposed methods against conventional CS, focusing on the visual fidelity of the reconstructed images. In the absence of fully sampled ground truth data, which is not feasible for clinical breath‐hold MRCP acquisitions, we used our respiratory‐triggered acquisitions as a surrogate reference. This choice was motivated by its widespread clinical use. We would like to note that the image quality of respiratory‐triggered MRCP is strongly dependent on the regularity of the breathing pattern, and irregular breathing patterns are the main reasons why scans need to be repeated in practice. As described in the section, *Dataset for zero‐shot learning*, we repeated the triggered acquisition for some volunteers until we were able to achieve a sufficiently regular breathing pattern that could serve as a reference for image quality.

A qualitative reader study was conducted using five evaluation criteria: overall image quality, sharpness, noise/SNR, aliasing artifacts, and regularization artifacts (over‐regularization). Three MRI experts (15, 18, and > 20 years of experience in MRI) independently evaluated 55 reconstructions derived from 11 subjects, comprising CS and four frozen/trainable zero‐shot learning configurations. For each reconstruction, both 3D source images and maximum intensity projection (MIP) images were provided for assessment. Scores were assigned using a Likert scale: 1 = poor, 2 = fair, 3 = good, and 4 = excellent, and analyzed on a per‐subject basis. Statistical comparisons between reconstruction methods were performed using two‐sided paired Wilcoxon signed‐rank tests, with p<0.05 considered statistically significant.

For the quantitative analysis, PSNR (dB) was computed from the retrospectively undersampled data at *R* = 25 against its corresponding breath‐hold acquisition at *R* = 6 reconstructed with $\ell_{1}$‐wavelet CS reconstruction.

The reconstruction time of zero‐shot learning was measured over the entire reconstruction pipeline, including preprocessing, training, and the final reconstruction.

## 3 Results

**FIGURE 2:**
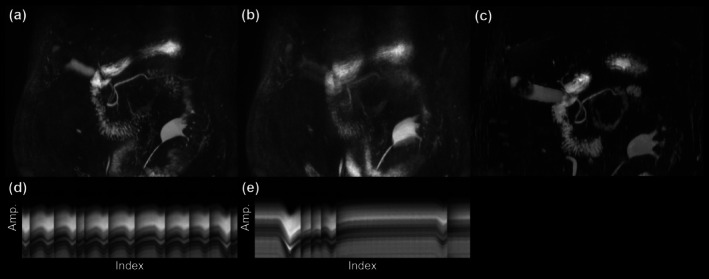
MIP views showing the impact of respiratory patterns on respiratory‐triggered MRCP acquisition (a, b) and a corresponding breath‐hold acquisition with 0/13 zero‐shot learning reconstruction of the same volunteer (c, volunteer #10). Subsets of the corresponding respiratory navigator signals are shown in (d) and (e). The *x*‐axis indicates signal sampling index of the acquired navigator, and the *y*‐axis indicates the acquired projections of the liver‐dome navigator.

**FIGURE 3:**
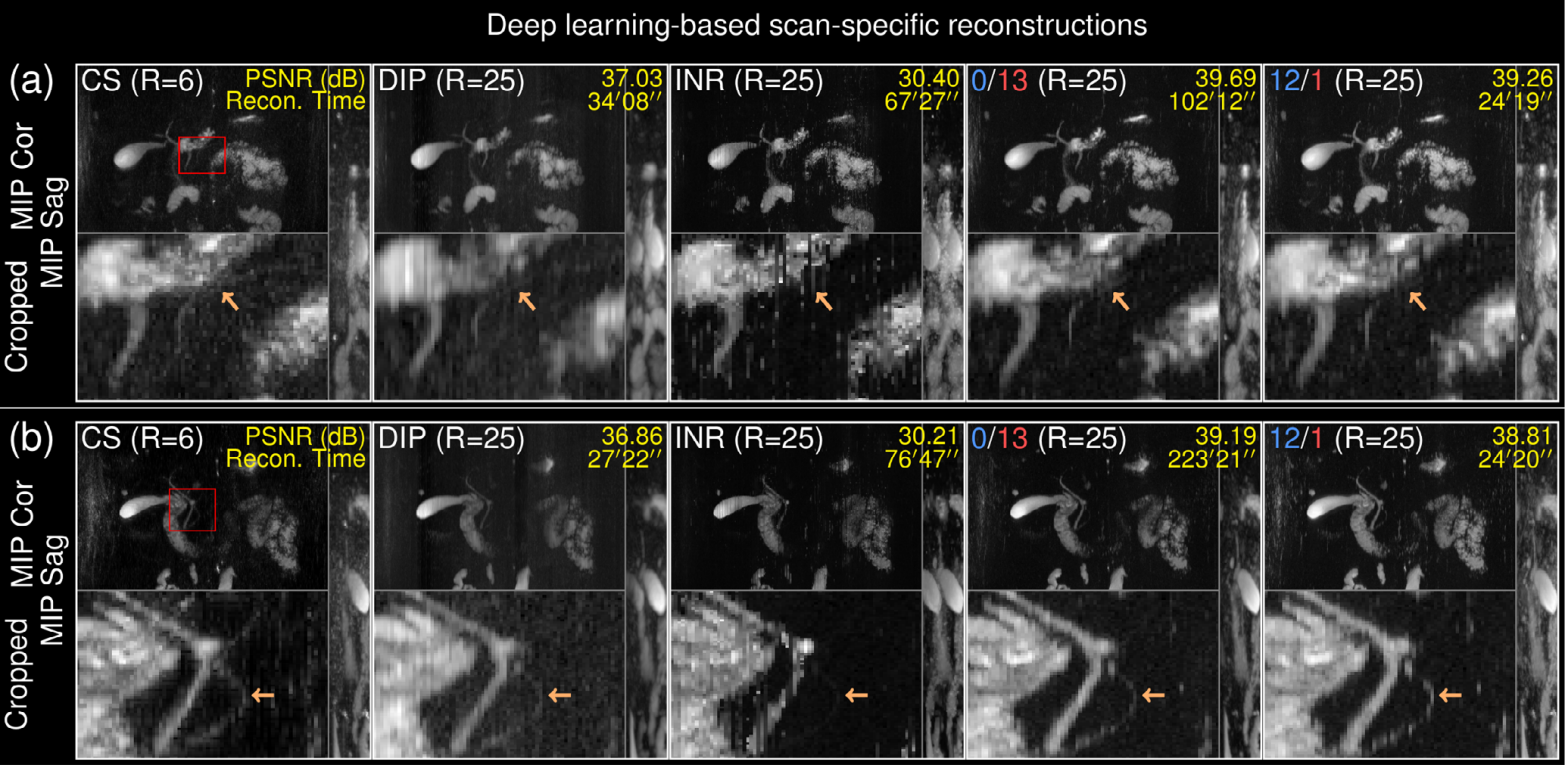
Results of deep learning‐based scan‐specific reconstructions on retrospectively undersampled acquisition from (a) a 33‐year‐old male (volunteer #01) and (b) a 46‐year‐old male (volunteer #02). CS, $\ell_{1}$‐wavelet compressed sensing; DIP, deep image prior; INR, implicit neural representation; zero‐shot learning using different frozen/trainable‐stage configurations, 0/13 and 12/1. Each block shows a breath‐hold acquisition at *R* = 6 and its retrospectively undersampling at *R* = 25. For each method, three visualizations are provided: a coronal maximum intensity projection (MIP, top left), a cropped coronal MIP focused on the region of interest (bottom left), and a sagittal MIP (right). The red box in the coronal MIP marks the ROI shown in the cropped view. Peak signal‐to‐noise ratio (PSNR) was computed against the original data at *R* = 6. “Recon. Time” indicates the reconstruction time. The orange arrows highlight regions with notable differences in ductal visibility across reconstructions.

Figure 2 shows a comparison of a successful respiratory‐triggered acquisition (Figure 2a), an unsuccessful respiratory‐triggered scan (Figure 2b), and a 14‐s breath‐hold acquisition (Figure 2c) for one of our volunteer scans (volunteer #10). The respiratory‐triggered acquisitions were reconstructed using GRAPPA, whereas the breath‐hold acquisition was reconstructed using zero‐shot learning with the 0/13 configuration. The corresponding respiratory patterns are shown in Figure 3d,e. These results show that successful respiratory‐triggering yields sharp and artifact‐free images. Irregular or shallow breathing introduced motion blurring and navigator delays, which resulted in degraded duct visibility and prolonged the scan time from 360‐s (regular breathing) to 587‐s (irregular breathing). In comparison, the 14‐s breath‐hold acquisition (Figure 2c) reduced motion artifacts in comparison to the failed triggering, but showed increased noise and reduced biliary visibility (e.g., pancreatic duct) in comparison to the successful triggering. Full respiratory traces of Figure 3d,e are available in Figure S2.

In Figure 3, retrospectively undersampled data were reconstructed using zero‐shot learning with two frozen/trainable‐stage configurations (0/13 and 12/1) and compared with DIP and INR reconstructions. PSNR was computed against the $\ell_{1}$‐wavelet CS at *R* = 6. For both datasets, DIP reconstructions exhibited amplified background noise and noticeable image blurring, despite achieving PSNRs of 37.03 dB/36.86 dB with reconstruction times of $34^{\prime }08^{\prime \prime }$ and $27^{\prime }22^{\prime \prime }$, respectively. In contrast, INR produced visually sharper images with reduced blurring, but yielded substantially lower PSNR values of 30.40 dB/30.21 dB and longer reconstruction times of $67^{\prime }27^{\prime \prime }$ and $76^{\prime }47^{\prime \prime }$ compared with DIP. Both zero‐shot configurations demonstrated reduced background noise and improved preservation of fine ductal structures relative to DIP and INR, resulting in the highest PSNR values among all compared methods.

**FIGURE 4:**
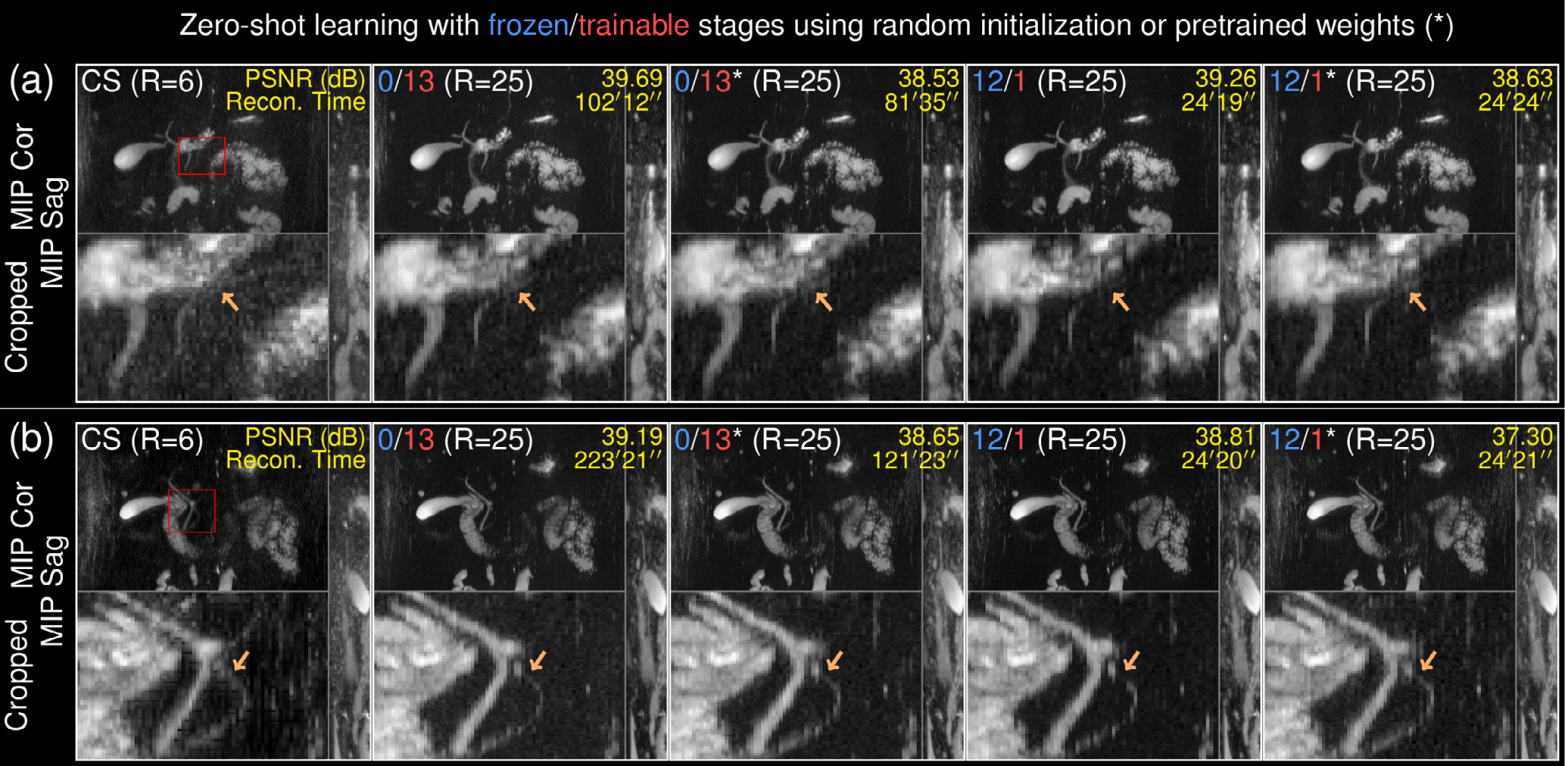
Reconstruction results of retrospectively undersampled acquisition from (a) a 33‐year‐old male (volunteer #01) and (b) a 46‐year‐old male (volunteer #02) using zero‐shot learning. Each block shows a breath‐hold acquisition at *R* = 6 and its retrospectively undersampling at R = 25, reconstructed using different frozen/trainable configurations (0/13 and 12/1). The asterisk (*) means models where the trainable stages were initialized with pretrained weights. For each method, three visualizations are provided: a coronal maximum intensity projection (MIP, top left), a cropped coronal MIP focused on the region of interest (bottom left), and a sagittal MIP (right). The red box in the coronal MIP marks the ROI shown in the cropped view. Peak signal‐to‐noise ratio (PSNR) was computed against $\ell_{1}$‐wavelet compressed sensing reconstruction on the original data at *R* = 6. “Recon. Time” indicates the reconstruction time. The orange arrows highlight regions with notable differences in ductal visibility across reconstructions.

Figure 4 shows representative reconstructions with the PSNR and reconstruction times of zero‐shot learning with different initialization strategies for the trainable stages for two retrospectively undersampled acquisitions. The orange arrows highlight regions with notable differences in ductal visibility across reconstructions. The reference for PSNR in this study was the breath‐hold MRCP at *R* = 6 reconstructed with $\ell_{1}$‐wavelet CS reconstruction. The asterisk (*) in the figure legend indicates that the network for the trainable stages is initialized with the pretrained weights. For zero‐shot learning with random initialization across both datasets, the 0/13 configuration achieved PSNRs of 39.69 dB/39.19 dB with reconstruction times of $102^{\prime }12^{\prime \prime }$/$223^{\prime }21^{\prime \prime }$, whereas the 12/1 configuration yielded comparable PSNRs of 39.26 dB/38.81 dB but reduced the reconstruction times to $24^{\prime }19^{\prime \prime }$/$24^{\prime }20^{\prime \prime }$. For zero‐shot learning with pretrained weight initialization, the 0/13* configuration produced PSNRs of 38.53 dB/38.65 dB with reconstruction times of $81^{\prime }35^{\prime \prime }$/$121^{\prime }23^{\prime \prime }$, while the 12/1* configuration achieved 38.63 dB/37.30 dB with substantially shorter reconstruction times of $24^{\prime }24^{\prime \prime }$/$24^{\prime }21^{\prime \prime }$.

**FIGURE 5:**
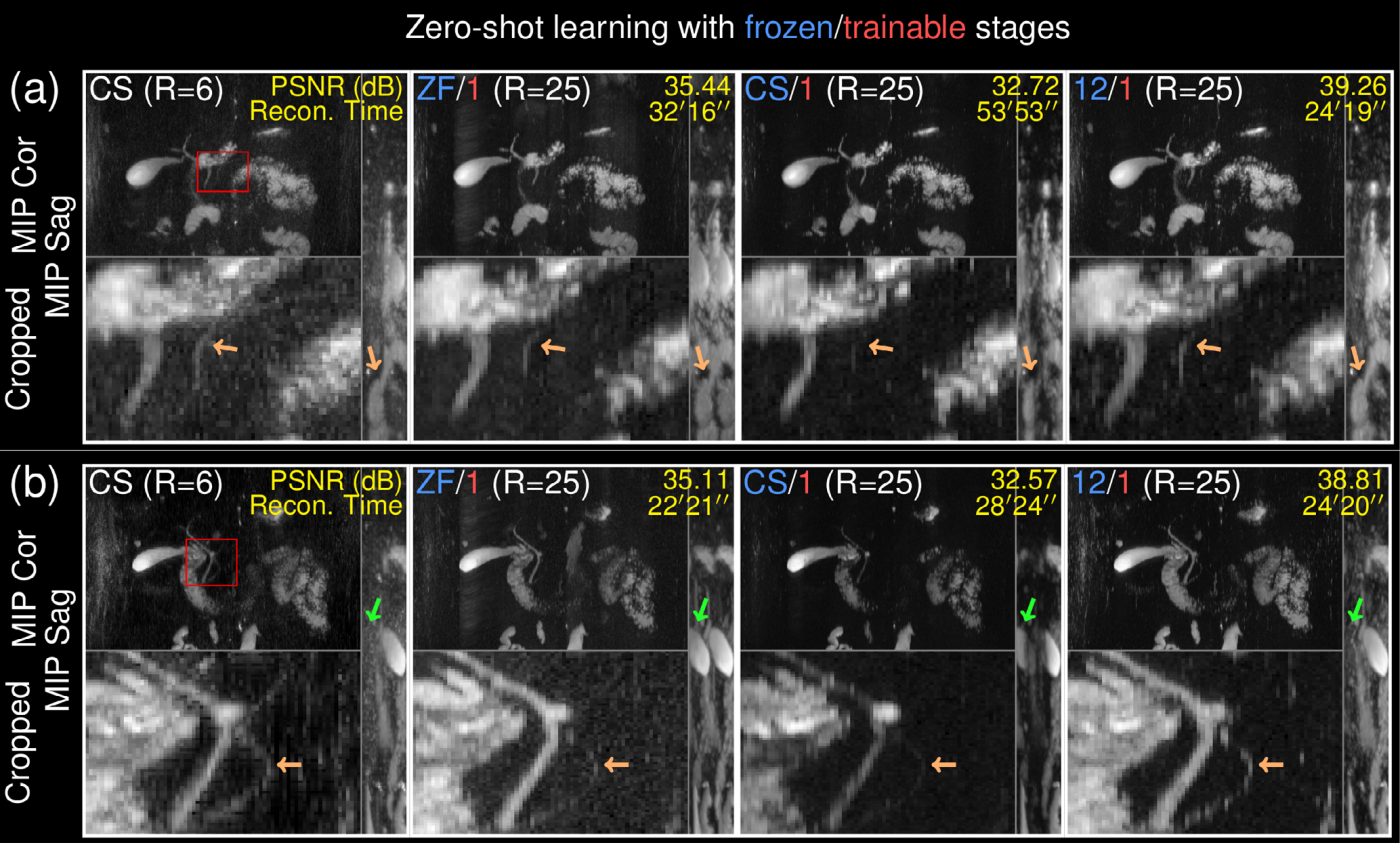
Reconstruction results of different initializations for the frozen stages of zero‐shot learning from (a) a 33‐year‐old male (volunteer #01) and (b) a 46‐year‐old male (volunteer #02). Each block shows a breath‐hold acquisition at *R* = 6 and its retrospectively undersampling at *R* = 25, reconstructed using zero‐filled reconstruction (ZF/1), $\ell_{1}$‐wavelet compressed sensing reconstruction (CS/1) and the 12‐stage pretrained network (12/1) for the frozen parts with a single trainable stage configuration. For each method, three visualizations are provided: a coronal maximum intensity projection (MIP, top left), a cropped coronal MIP focused on the region of interest (bottom left), and a sagittal MIP (right). The red box in the coronal MIP marks the ROI shown in the cropped view. Peak signal‐to‐noise ratio (PSNR) was computed against the original data at *R* = 6. “Recon. Time” indicates the reconstruction time. The orange arrows highlight regions with notable differences in anatomical details, and the green arrows point to the aliasing artifacts across reconstructions.

Figure 5 depicts reconstruction results obtained using three different initialization strategies for the frozen stages: zero‐filled reconstruction (ZF/1), $\ell_{1}$‐wavelet CS reconstruction (CS/1), and n‐stage pretrained network (12/1). In all configurations, a single appended stage was randomly initialized and optimized in a zero‐shot manner. Two retrospectively undersampled acquisitions were reconstructed. The orange arrows indicate regions with visible differences in anatomical details, and the green arrows point to aliasing artifacts along the partition‐encoding direction particularly for CS/1 in Figure 5b. Across both datasets, the 12/1 zero‐shot configurations yielded higher PSNR values (39.26 dB/38.81 dB) compared with either ZF/1 (35.44 dB/35.11 dB) and CS/1 (32.72 dB/32.57 dB). The reconstruction times with 12 frozen stages ($24^{\prime }19^{\prime \prime }$/$24^{\prime }20^{\prime \prime }$) were also shorter than those obtained with either the zero‐filled initialization ($32^{\prime }16^{\prime \prime }$/$22^{\prime }21^{\prime \prime }$) and the CS initialization ($53^{\prime }53^{\prime \prime }$/$28^{\prime }24^{\prime \prime }$). Particularly, the ZF/1 configuration could not suppress the aliasing artifacts across undersampling directions.

**FIGURE 6:**
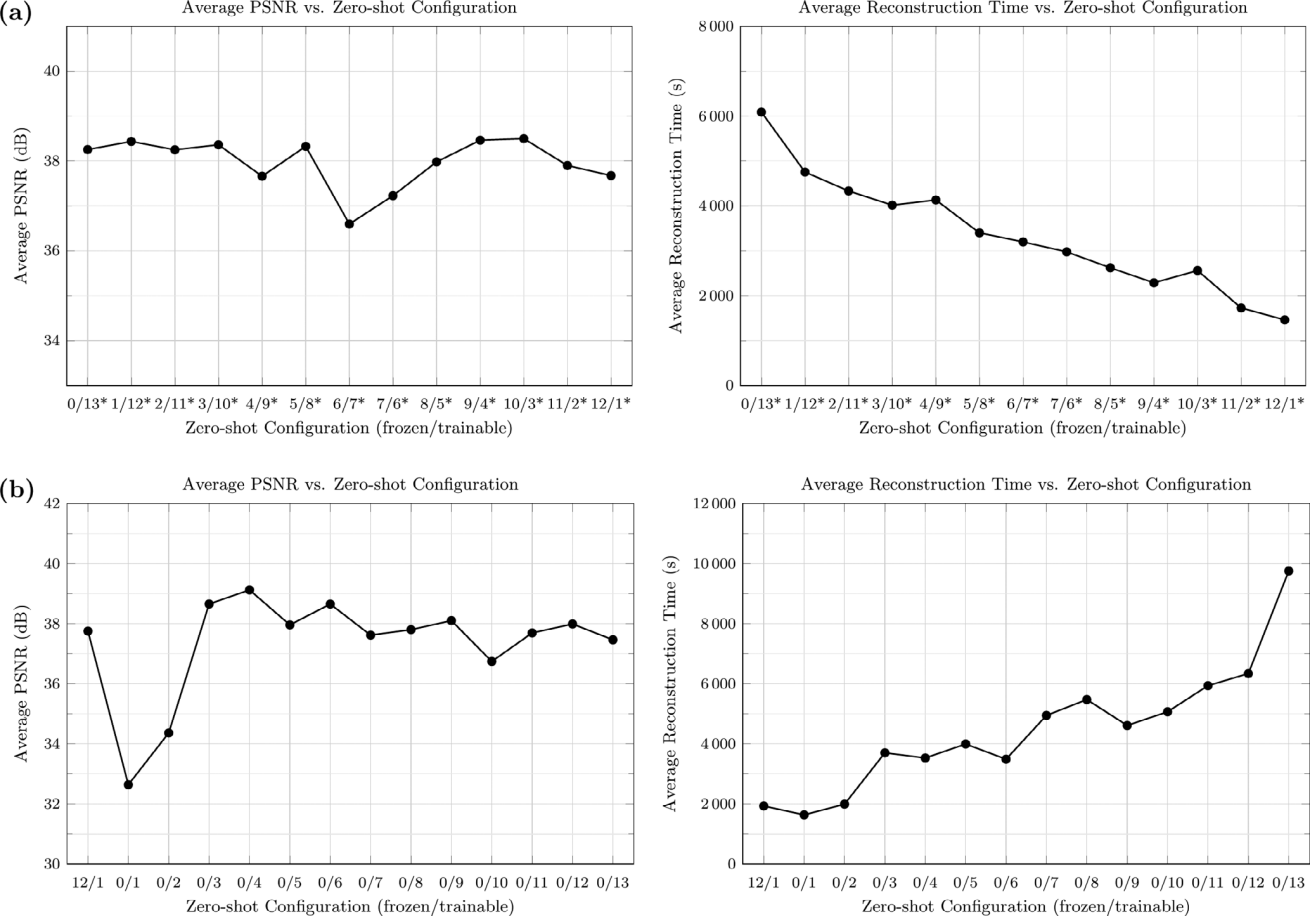
Lightweight zero‐shot learning strategies illustrating performance and training time when (a) varying the number of n frozen and m trainable stages under the constraint n+m=13; and (b) fixing the number of frozen stages to zero (n=0) and varying the number of m trainable stages. For each (a) and (b), the left plots show average PSNR in dB and the right plots show average training time in seconds. The asterisk (*) indicates that the trainable stages were initialized with the corresponding pretrained network. Each configuration specifies the number of frozen and trainable stages in the zero‐shot model. Results are averaged across two retrospectively undersampled datasets at *R* = 25. PSNR was calculated against the original acquisition at *R* = 6 using $\ell_{1}$‐wavelet compressed sensing reconstruction.

Figure 6 summarizes the average PSNR and reconstruction times from two retrospectively undersampled datasets across different zero‐shot configurations. In Figure 6a, the number of frozen (n) and trainable (m) stages was varied under the constraint n+m=13, spanning configurations from 0/13* to 12/1*. The asterisk (*) indicates that the trainable stages were initialized using the corresponding pretrained weights. In Figure 6b, the number of frozen stages was fixed to zero while the number of trainable stages (m) was varied.

In Figure 6a, PSNR remained largely stable across configurations (37.97 ± 0.54 dB, median: 38.25 dB), despite substantial changes in the frozen/trainable ratio. In contrast, reconstruction time decreased approximately linearly with increasing numbers of frozen stages, demonstrating that freezing early stages provides substantial computational savings without compromising reconstruction fidelity.

In Figure 6b, PSNR increased rapidly with the first few trainable stages and plateaued after approximately three stages, whereas reconstruction time continued to increase monotonically with additional trainable stages. Notably, the 12/1 configuration achieved a PSNR comparable to the models containing more than three trainable stages, while requiring least reconstruction time, highlighting the efficiency of the pretrained frozen stages.

**FIGURE 7:**
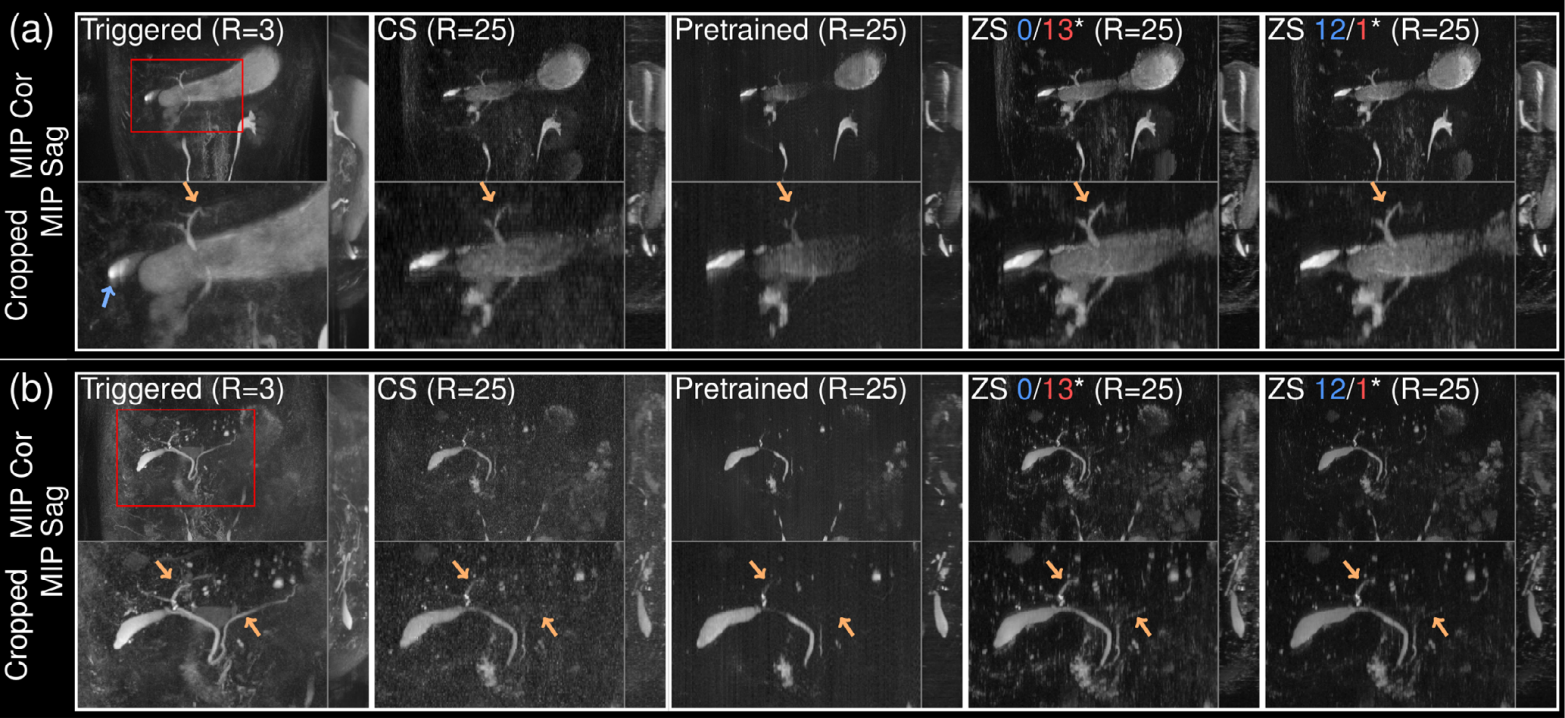
Reconstruction results from (a) a 28‐year‐old male (volunteer #07) and (b) a 64‐year‐old male (volunteer #08). Each reconstruction block shows results from a triggered acquisition (Triggered) and a 14 s breath‐hold acquisition reconstructed with compressed sensing (CS), a pretrained reconstruction model used for the frozen stages of ZS 12/1* (pretrained), zero‐shot learning with frozen/trainable configurations of 0/13* and 12/1*, respectively. The asterisk (*) indicates that the network for the m‐trainable stages is initialized with the m‐stage pretrained weights. For each method, three visualizations are provided: a coronal maximum intensity projection (MIP, top left), a cropped coronal MIP focused on the region of interest (bottom left), and a sagittal MIP (right). The red box in the coronal MIP marks the ROI shown in the cropped view. The orange arrows highlight regions with notable differences in ductal visibility across reconstructions. The blue arrow indicates motion artifacts in the triggered acquisition.

Figure 7 shows reconstruction results from a 28‐year‐old male (volunteer #07, Figure 7a) and a 64‐year‐old male (volunteer #08, Figure 7b). The figure compares the image quality of triggered acquisitions to 14 s breath‐hold acquisitions reconstructed with CS, a pretrained reconstruction model used for the frozen stages of ZS 12/1*, zero‐shot learning with frozen/trainable configurations of 0/13* and 12/1*, respectively. Each reconstruction includes multiple visualizations (MIP Coronal, Cropped, and MIP Sagittal). The asterisk (*) indicates that the network for the trainable stages is initialized with the pretrained weights. With the exception of residual motion artifacts (highlighted by the blue arrow in Figure 7a), no breath‐hold acquisition reaches the image quality of the (successful) triggered acquisition. However, ductal visibility (highlighted by orange arrows), contrast to noise ratio and absence of aliasing artifacts are superior for both zero‐shot methods in comparison to CS and the pretrained reconstruction model. While 12/1* zero‐shot training strategy shows a minor reduction in fine structural details relative to 0/13* zero‐shot training, it still outperformed CS in terms of contrast to noise ratio and duct delineation. Full reconstruction results for additional subjects are provided in Figures S3–S10.

Table 2 shows reconstruction times of zero‐shot training with different configurations of frozen/trainable stages for all 11 volunteers. The 0/13 configuration required $136^{\prime }16^{\prime \prime }$ with random initialization and $78^{\prime }46^{\prime \prime }$ with pretrained initialization, whereas the 12/1 configuration reduced the reconstruction times to $20^{\prime }25^{\prime \prime }$ and $18^{\prime }19^{\prime \prime }$, respectively. This corresponds to 6.7‐ and 4.3‐fold speedup in reconstruction time for the 12/1 configuration relative to 0/13 for each random and pretrained initialization, respectively.

**TABLE 2:** Reconstruction times of zero‐shot learning with different configurations of frozen/trainable stages for all 11 volunteers in this study.

Zero‐shot configurations (frozen/trainable)			
Volunteer	0/13	0/13^a^	12/1	12/1^a^
#01	$122^{\prime }14^{\prime \prime }$	$077^{\prime }39^{\prime \prime }$	$19^{\prime }21^{\prime \prime }$	$19^{\prime }33^{\prime \prime }$
#02	$131^{\prime }31^{\prime \prime }$	$074^{\prime }00^{\prime \prime }$	$22^{\prime }08^{\prime \prime }$	$15^{\prime }32^{\prime \prime }$
#03	$190^{\prime }34^{\prime \prime }$	$077^{\prime }47^{\prime \prime }$	$19^{\prime }22^{\prime \prime }$	$19^{\prime }15^{\prime \prime }$
#04	$203^{\prime }47^{\prime \prime }$	$112^{\prime }10^{\prime \prime }$	$17^{\prime }53^{\prime \prime }$	$17^{\prime }31^{\prime \prime }$
#05	$115^{\prime }29^{\prime \prime }$	$077^{\prime }03^{\prime \prime }$	$19^{\prime }05^{\prime \prime }$	$19^{\prime }18^{\prime \prime }$
#06	$107^{\prime }48^{\prime \prime }$	$075^{\prime }51^{\prime \prime }$	$18^{\prime }02^{\prime \prime }$	$17^{\prime }54^{\prime \prime }$
#07	$129^{\prime }07^{\prime \prime }$	$075^{\prime }26^{\prime \prime }$	$17^{\prime }24^{\prime \prime }$	$17^{\prime }36^{\prime \prime }$
#08	$121^{\prime }58^{\prime \prime }$	$069^{\prime }22^{\prime \prime }$	$19^{\prime }04^{\prime \prime }$	$19^{\prime }35^{\prime \prime }$
#09	$121^{\prime }28^{\prime \prime }$	$076^{\prime }31^{\prime \prime }$	$19^{\prime }45^{\prime \prime }$	$19^{\prime }27^{\prime \prime }$
#10	$083^{\prime }47^{\prime \prime }$	$074^{\prime }15^{\prime \prime }$	$24^{\prime }57^{\prime \prime }$	$16^{\prime }48^{\prime \prime }$
#11	$171^{\prime }22^{\prime \prime }$	$076^{\prime }28^{\prime \prime }$	$27^{\prime }37^{\prime \prime }$	$19^{\prime }00^{\prime \prime }$
Average	$136^{\prime }16^{\prime \prime }$	$078^{\prime }46^{\prime \prime }$	$20^{\prime }25^{\prime \prime }$	$18^{\prime }19^{\prime \prime }$

^a^ The network for the m‐trainable stages is initialized with the m‐stage pretrained network.

**FIGURE 8:**
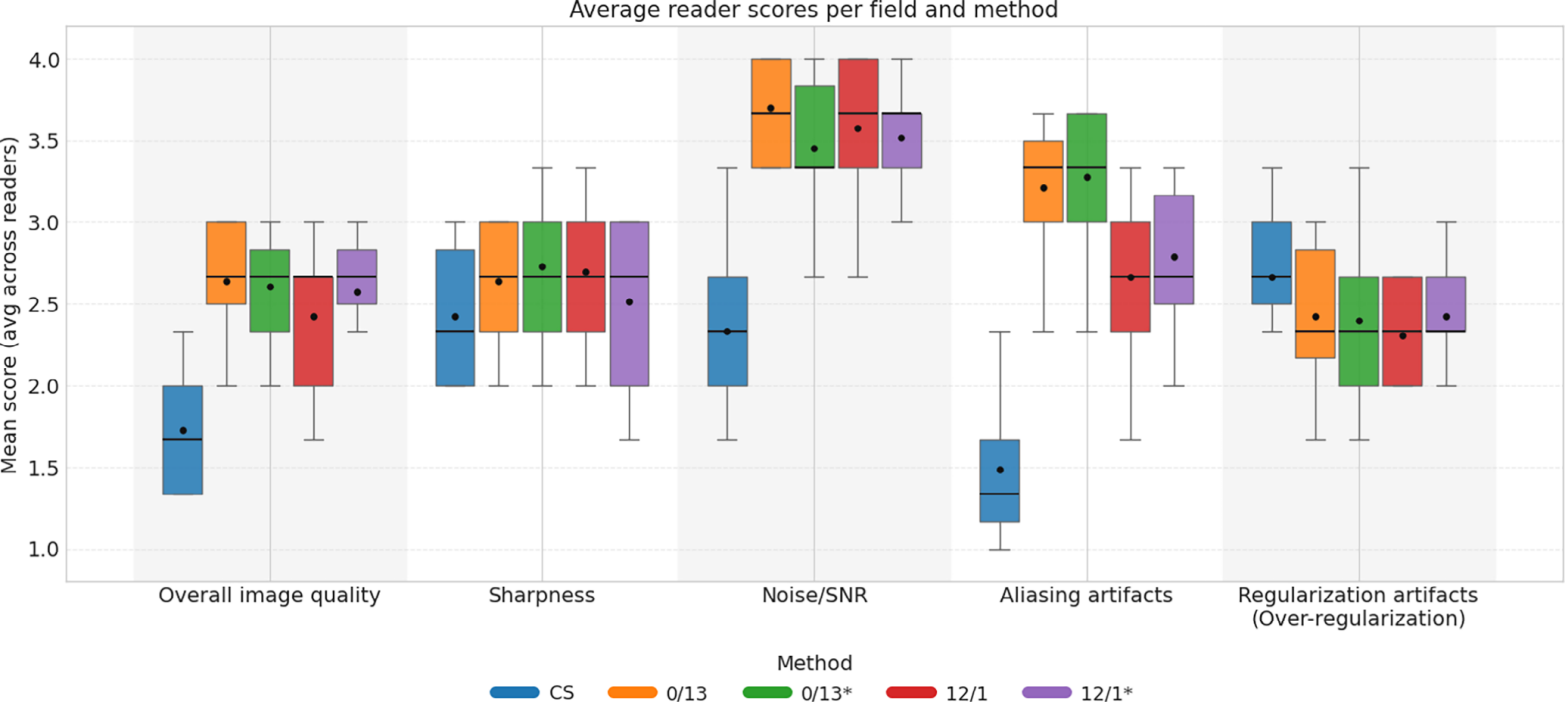
Box plots of reader study scores for compressed sensing (CS) and four frozen/trainable zero‐shot learning configurations. Five qualitative assessment criteria are shown on the x‐axis: Overall image quality, sharpness, noise/SNR, aliasing artifacts, and regularization artifacts (over‐regularization). Scores represent subject‐wise means averaged across three readers. Boxes indicate the interquartile range (IQR), the central line denotes the median, whiskers represent the full range, and black dots indicate the mean. Reconstruction methods are color‐coded as follows: CS (blue), 0/13 (orange), 0/13* (green), 12/1 (red), and 12/1* (purple). The asterisk (*) indicates that the trainable stages were initialized using pretrained weights.

Figure 8 shows box plots summarizing the qualitative reader study scores across reconstruction methods, including CS and four frozen/trainable zero‐shot configurations. For each reconstruction method, scores were averaged across three readers and subsequently summarized over 11 subjects. Compared with CS, all zero‐shot configurations demonstrated significantly higher scores in overall image quality, noise/SNR, and aliasing artifacts. The 0/13 configurations tended to yield higher overall image quality scores than the 12/1 configurations, whereas 12/1 configurations showed comparable performance to 0/13 in noise/SNR and aliasing artifacts. For sharpness, zero‐shot reconstructions achieved higher mean scores than CS; however, these differences were not statistically significant. In contrast, CS received higher scores for regularization artifacts than all zero‐shot configurations, meaning that CS visually presents more natural image quality. This is true because the regularization parameter for CS was defined to balance data fidelity and noise suppression, while those parameters for zero‐shot reconstruction were learned during training, potentially leading to over‐regularization. Numerical statistics are provided in Table S1.

## 4 Discussion

This work investigates the technical feasibility of reconstructing highly accelerated 3D breath‐hold MRCP using zero‐shot self‐supervised learning. By combining a hybrid undersampling strategy with scan‐specific reconstruction, image quality can be substantially improved at an acceleration factor of *R* = 25 for a 14‐s breath‐hold, relative to conventional CS. In addition, we introduce a partially trainable zero‐shot framework that significantly reduces reconstruction time while preserving image fidelity, addressing a key practical limitation of scan‐specific learning methods.

In the absence of a fully sampled ground truth, we used respiratory‐triggered acquisitions as the reference for image quality in this study. While none of our breath‐hold acquisitions reached the image quality of the triggered acquisition in the case of regular breathing patterns, we noticed that even young healthy volunteers reported discomfort from the prolonged and inconsistent acquisition times. In four volunteers, we had to repeat the respiratory‐triggered acquisitions to achieve sufficient image quality.

While DIP and INR represent scan‐specific reconstruction strategies, their optimization differs fundamentally from the proposed approach. In this study, all methods were implemented using a consistent forward model, identical coil sensitivity maps, and comparable *k*‐space data fidelity losses (see Supporting Information Section B). Under these controlled conditions, the primary distinction lies in the explicit self‐supervised data consistency enforcement in the zero‐shot framework, whereas DIP and INR rely on implicit regularization through network parameterization. The observed reduction in noise amplification and improved preservation of ductal structures therefore suggests that the performance gains are largely attributable to the self‐supervised data consistency formulation rather than architectural differences alone. We emphasize that the goal of this comparison is not to exhaustively benchmark scan‐specific reconstruction methods, but to provide representative baselines illustrating the behavior of implicit‐prior‐based approaches under highly accelerated MRCP conditions.

Our results demonstrated superior image quality of image reconstruction using zero‐shot learning. Conventional CS resulted in poor depiction of ductal structures at such a high acceleration rate. Using a pretrained model trained with retrospective subsampling of triggered acquisitions also led to inferior image quality. Several systematic differences from our previous study [27] likely contributed to this performance discrepancy. These include a substantially higher acceleration factor (*R* = 25 vs. *R* = 6), the additional use of partial Fourier acquisition, and different sampling strategies (incoherent vs. equidistant).

For the quantitative evaluation, 6‐fold accelerated breath‐hold acquisitions were retrospectively undersampled to *R* = 25 by applying the *R* = 25 sampling mask, exploiting the fact that the *R* = 25 sampling pattern is a subset of the *R* = 6 acquisition, and PSNR values were computed against the corresponding *R* = 6 reconstructions. As six‐fold accelerated breath‐hold data were acquired from healthy volunteers who could manage long breath‐hold, we assumed those acquisitions a constant motion state, such that the retrospective experiments primarily reflect reconstruction‐related effects rather than motion variability. Since the acceleration factor of *R* = 6 already leads to noticeable noise amplification [45], and this effect is particularly pronounced in MRCP due to its inherently sparse signal characteristics, quantitative image quality assessment in this setting is challenging [27]. To avoid overinterpretation of similarity metrics under these conditions, we limited the quantitative evaluation to PSNR and complemented the quantitative analysis with the qualitative reader study.

The qualitative reader study demonstrated that zero‐shot reconstruction consistently improved perceived image quality compared with CS, particularly in overall image quality, noise suppression, and reduction of aliasing artifacts. These findings suggest that subject‐specific optimization enhances image quality efficiently. Although improvements in sharpness did not reach statistical significance, zero‐shot configurations achieved comparable or higher mean scores than CS, indicating that noise reduction was not achieved at the expense of structural detail. Overall, the reader study confirms that zero‐shot reconstruction provides perceptually meaningful benefits over conventional CS.

Several strategies can be considered to reduce the training time of zero‐shot learning. One effective approach is the proposed partially trainable zero‐shot configuration, in which only the backpropagation depth varies with the number of trainable stages. Another alternative strategy is to reduce the total number of unrolled stages by training frozen/trainable stage configurations of the form 0/m (1≤m≤13). Together, these results demonstrate that substantial reductions in reconstruction time can be achieved without largely sacrificing reconstruction fidelity when leveraging pretrained representations.

As a further alternative, we investigated truncating the training of the fully trainable 0/13 configuration to match the reconstruction time required by the 12/1 configuration. However, as shown in Figure S11, this approach did not yield reliable convergence. The required training duration varied considerably across subjects, leading to inconsistent reconstruction quality and making it impractical to define a fixed stopping criterion a priori.

Two types of artifacts were observed in our reconstruction results. First, since we decoupled the fully sampled readout direction and performed 2D reconstruction across the two phase‐encoding directions, discontinuities between adjacent readout planes occasionally appeared, especially in the background region. Figure S12 shows the comparison of 2D and 3D reconstructions demonstrating that discontinuities are not observed in 3D reconstruction. Considering the fact that joint 3D reconstruction performs more robustly than 2D reconstruction and the proposed lightweight partially trainable strategy, joint 3D zero‐shot reconstruction can be investigated in future works to further improve the performance [46, 47, 48].

Second, the sampling pattern contained equidistance undersampling along the phase‐encoding (*R* = 3) and partition‐encoding (*R* = 2) directions. Given the relatively small number of partition‐encoding lines, the acceleration factor of *R* = 2 in this direction led to residual aliasing in the partition dimension. This artifact is shown in Figures 5b and S13 with green arrows. The effect of the sampling geometry is analyzed in Figure S14. This hybrid sampling strategy was selected because, at a high acceleration factor of *R* = 25, pure Poisson‐disk undersampling resulted in increased blurring, whereas the inclusion of equidistant components improved high‐frequency coverage and preserved structural sharpness. Although CS reconstructions exhibited coherent aliasing along the partition direction, the zero‐shot reconstructions effectively suppressed these artifacts during scan‐specific refinement. A quantitative analysis for these artifacts was not performed, as fully sampled reference data are not available for breath‐hold MRCP, and the retrospectively undersampled reference (*R* = 6) already exhibits noise amplification, limiting the reliability of quantitative metrics for this purpose.

In our different initialization approaches for the trainable stages, pretrained initialization consistently converged faster than random. For the 0/13 configurations, the impact of initialization was minimal in both quantitative metrics and the visual appearance. In contrast, for the 12/1 configurations, pretrained‐weight initialization resulted in better image quality, particularly in preserving the visibility of the pancreatic ducts (orange arrows in Figure 4). We additionally found that initialization of the frozen stages played a big role in the partially trainable strategy as the output of the frozen stages served an initial input ($x^{n}$ in Figure 1b) to the trainable stages.

We would like to note that although partial Fourier acquisition was employed during data acquisition, no partial Fourier reconstruction techniques were applied. Future work may explore the integration of partial Fourier methods into deep learning reconstructions [49] to further enhance image quality. Moreover, subject‐specific hyperparameter adjustment, such as learning rate tuning, may be necessary to optimize reconstruction performance across different cases. As an example, the learning rate for volunteer #11 was reduced to 0.0001 since the initial value of 0.0003 caused optimization instability and prevented convergence, whereas the default value was optimal to the other cases. With the reduced learning rate, zero‐shot reconstruction for volunteer #11 achieved image quality and reconstruction time comparable to the other zero‐shot models.

The main limitation of zero‐shot learning is the long computation time. This motivated the development of the partial trainable zero‐shot training strategy, which reduces backpropagation depth by splitting a total number of stages into frozen and trainable stages. While this approach substantially decreases training time compared to conventional zero‐shot learning, we acknowledge that the overall reconstruction time still remains longer than that of other non‐data‐specific deep learning‐based methods. Various deep learning reconstruction models could be used in this framework. In this study, we selected SSDU^28^ as the backbone due to its architectural similarity to zero‐shot learning and its self‐supervised formulation, which makes it particularly well suited for MRCP where fully sampled data are unavailable [27].

Even though the participants of our study are from a representative age group (the oldest subject being 83 years old), one limitation is the exclusive use of healthy volunteer data. Therefore, our results do not allow for generalizing our findings to pathologic cases. Also, the distribution of regular and irregular breathing patterns as well as the consistency of breath‐holds may vary in a clinical population with sick patients [20]. Furthermore, the image quality assessment was based solely on visual inspection by the authors, without any involvement of radiologists or a radiologist‐led reader study. Future work should validate the clinical applicability of zero‐shot methods using patient datasets and expert diagnostic assessments.

Lastly, since the proposed partially trainable strategy is not inherently sequence‐specific, it may be extendable to other unrolled scan‐specific self‐supervised reconstruction settings, although this was not evaluated in the current study.

## 5 Conclusion

The goal of our study was to investigate the technical feasibility of shortening breath‐hold MRCP acquisitions through highly accelerated reconstruction methods. Our results demonstrate that zero‐shot self‐supervised learning achieves higher reconstruction fidelity than conventional CS and pretrained reconstruction models at acceleration factors required for 14‐s breath‐hold acquisitions. These findings suggest that zero‐shot learning is a promising reconstruction approach when large training datasets are unavailable.

We also introduce a zero‐shot training strategy with reduced backpropagation depth that reduced the reconstruction time from $136^{\prime }16^{\prime \prime }$ to $20^{\prime }25^{\prime \prime }$ for random initialization and from $78^{\prime }46^{\prime \prime }$ to $18^{\prime }19^{\prime \prime }$ for pretrained‐weight initialization, with only a minor trade‐off in reconstruction quality.

## Funding

This work was supported by the German Research Foundation (DFG) (projects 513220538, 512819079, 556079651, 500888779, and 440719683) and the Friedrich‐Alexander‐University Erlangen‐Nuremberg (FAU) (5011000001652). NHR funding was provided by the Federal Ministry of Education and Research (Bundesministerium für Bildung und Forschung – BMBF) and the Free State of Bavaria participating in accordance with the resolutions of the Joint Science Conference (Gemeinsame Wissenschaftskonferenz – GWK) for the national high‐performance computing at universities.

## Conflicts of Interest

J.K. receives a PhD stipend from Siemens Healthineers AG. M.D.N. is employed by Siemens Healthineers AG. F.K. receives patent royalties for deep learning image reconstruction and research support from Siemens Healthineers AG, has stock options from Subtle Medical, and is a consultant for Imaginostics.

## Supporting information


**Figure S1:** Undersampling pattern for breath‐hold MRCP, combining 2D Poisson‐disk incoherent undersampling with partial Fourier undersampling, leading to a total acceleration factor of *R* = 25 and 14‐s breath‐hold scans. The *x*‐ and *y*‐axis correspond to the in‐plane phase encoding and partition encoding directions, respectively. The fully sampled readout (frequency encoding) direction is orthogonal to the phase‐partition encoding plane (i.e., through‐plane).
**Figure S2:** Full respiratory navigator signals corresponding to Figure 3d,e are shown in (a) and (b), respectively. Acquisition time was 360 s for (a) and 587 s for (b). Red boxes indicate the regions shown in the zoomed‐in views in Figure 3. The *x*‐axis represents the signal sampling index, and the *y*‐axis shows acquired projections of the liver‐dome navigator.
**Figure S3:** Reconstruction results from a 33‐year‐old male (volunteer #01). Reconstruction methods include a triggered acquisition (Triggered) and a 14 s breath‐hold acquisition reconstructed with compressed sensing (CS), a pretrained reconstruction model used for the frozen stages of ZS 12/1* (Pretrained), zero‐shot learning with frozen/trainable configurations of 0/13 and 12/1, respectively. The asterisk (*) indicates that the network for the trainable stages is initialized with the pretrained weights. For each method, three visualizations are provided: a coronal maximum intensity projection (MIP, top left), a cropped coronal MIP focused on the region of interest (bottom left), and a sagittal MIP (right). The red box in the coronal MIP marks the ROI shown in the cropped view.
**Figure S4:** Reconstruction results from a 27‐year‐old female (volunteer #03). Reconstruction methods include a triggered acquisition (Triggered) and a 14 s breath‐hold acquisition reconstructed with compressed sensing (CS), a pretrained reconstruction model used for the frozen stages of ZS 12/1* (Pretrained), zero‐shot learning with frozen/trainable configurations of 0/13 and 12/1, respectively. The asterisk (*) indicates that the network for the trainable stages is initialized with the pretrained weights. For each method, three visualizations are provided: a coronal maximum intensity projection (MIP, top left), a cropped coronal MIP focused on the region of interest (bottom left), and a sagittal MIP (right). The red box in the coronal MIP marks the ROI shown in the cropped view.
**Figure S5:** Reconstruction results from a 27‐year‐old male (volunteer #04). Reconstruction methods include a triggered acquisition (Triggered) and a 14 s breath‐hold acquisition reconstructed with compressed sensing (CS), a pretrained reconstruction model used for the frozen stages of ZS 12/1* (Pretrained), zero‐shot learning with frozen/trainable configurations of 0/13 and 12/1, respectively. The asterisk (*) indicates that the network for the trainable stages is initialized with the pretrained weights. For each method, three visualizations are provided: a coronal maximum intensity projection (MIP, top left), a cropped coronal MIP focused on the region of interest (bottom left), and a sagittal MIP (right). The red box in the coronal MIP marks the ROI shown in the cropped view.
**Figure S6:** Reconstruction results from a 29‐year‐old male (volunteer #05). Reconstruction methods include a triggered acquisition (Triggered) and a 14 s breath‐hold acquisition reconstructed with compressed sensing (CS), a pretrained reconstruction model used for the frozen stages of ZS 12/1* (Pretrained), zero‐shot learning with frozen/trainable configurations of 0/13 and 12/1, respectively. The asterisk (*) indicates that the network for the trainable stages is initialized with the pretrained weights. For each method, three visualizations are provided: a coronal maximum intensity projection (MIP, top left), a cropped coronal MIP focused on the region of interest (bottom left), and a sagittal MIP (right). The red box in the coronal MIP marks the ROI shown in the cropped view.
**Figure S7:** Reconstruction results from a 28‐year‐old male (volunteer #06). Reconstruction methods include a triggered acquisition (Triggered) and a 14 s breath‐hold acquisition reconstructed with compressed sensing (CS), a pretrained reconstruction model used for the frozen stages of ZS 12/1* (Pretrained), zero‐shot learning with frozen/trainable configurations of 0/13 and 12/1, respectively. The asterisk (*) indicates that the network for the trainable stages is initialized with the pretrained weights. For each method, three visualizations are provided: a coronal maximum intensity projection (MIP, top left), a cropped coronal MIP focused on the region of interest (bottom left), and a sagittal MIP (right). The red box in the coronal MIP marks the ROI shown in the cropped view.
**Figure S8:** Reconstruction results from a 46‐year‐old female (volunteer #09). Reconstruction methods include a triggered acquisition (Triggered) and a 14 s breath‐hold acquisition reconstructed with compressed sensing (CS), a pretrained reconstruction model used for the frozen stages of ZS 12/1* (Pretrained), zero‐shot learning with frozen/trainable configurations of 0/13 and 12/1, respectively. The asterisk (*) indicates that the network for the trainable stages is initialized with the pretrained weights. For each method, three visualizations are provided: a coronal maximum intensity projection (MIP, top left), a cropped coronal MIP focused on the region of interest (bottom left), and a sagittal MIP (right). The red box in the coronal MIP marks the ROI shown in the cropped view.
**Figure S9:** Reconstruction results from a 68‐year‐old male (volunteer #10). Reconstruction methods include a triggered acquisition (Triggered) and a 14 s breath‐hold acquisition reconstructed with compressed sensing (CS), a pretrained reconstruction model used for the frozen stages of ZS 12/1* (Pretrained), zero‐shot learning with frozen/trainable configurations of 0/13 and 12/1, respectively. The asterisk (*) indicates that the network for the trainable stages is initialized with the pretrained weights. For each method, three visualizations are provided: a coronal maximum intensity projection (MIP, top left), a cropped coronal MIP focused on the region of interest (bottom left), and a sagittal MIP (right). The red box in the coronal MIP marks the ROI shown in the cropped view.
**Figure S10:** Reconstruction results from an 86‐year‐old male (volunteer #11). Reconstruction methods include a triggered acquisition (Triggered) and a 14 s breath‐hold acquisition reconstructed with compressed sensing (CS), a pretrained reconstruction model used for the frozen stages of ZS 12/1* (Pretrained), zero‐shot learning with frozen/trainable configurations of 0/13 and 12/1, respectively. The asterisk (*) indicates that the network for the trainable stages is initialized with the pretrained weights. For each method, three visualizations are provided: a coronal maximum intensity projection (MIP, top left), a cropped coronal MIP focused on the region of interest (bottom left), and a sagittal MIP (right). The red box in the coronal MIP marks the ROI shown in the cropped view.
**Figure S11:** Reconstruction results of retrospectively undersampled acquisition from (a) a 33‐year‐old male (volunteer #01) and (b) a 46‐year‐old male (volunteer #02) using zero‐shot learning. Each block shows a breath‐hold acquisition at *R* = 6 and its retrospectively undersampling at *R* = 25, reconstructed using different frozen/trainable configurations: middle left, 0/13 with truncated reconstruction time; middle right, fully converged 0/13; and rightmost, 12/1. For each method, three visualizations are provided: a coronal maximum intensity projection (MIP, top left), a cropped coronal MIP focused on the region of interest (bottom left), and a sagittal MIP (right). The red box in the coronal MIP marks the ROI shown in the cropped view. Peak signal‐to‐noise ratio (PSNR) was computed against the original data at *R* = 6. “Recon. Time” indicates the reconstruction time. The orange arrows highlight regions with notable differences in ductal visibility across reconstructions.
**Figure S12:** Comparison of readout‐direction continuity between 2D and 3D reconstruction strategies. CS 2D denotes a slice‐wise compressed sensing reconstruction and CS 3D denotes a joint 3D compressed sensing reconstruction. For CS 3D, coil sensitivity maps are estimated from decoupled 2D data and stacked into a 3D volume using spatial regularization along the readout direction, whereas CS 2D uses independent 2D coil sensitivity maps per readout index.
**Figure S13:** Reconstruction results from a 33‐year‐old male (volunteer #01), demonstrating aliasing artifacts along the slice encoding direction in the sagittal MIP view. Each reconstruction block shows results from a triggered acquisition (Triggered) and a 14 s breath‐hold acquisition reconstructed with compressed sensing (CS), a pretrained reconstruction model used for the frozen stages of ZS 12/1* (Pretrained), zero‐shot learning with frozen/trainable configurations of 0/13* and 12/1*, respectively. The asterisk (*) indicates that the network for the trainable stages is initialized with the pretrained weights. Green arrows indicate areas where aliasing artifacts are observed.
**Figure S14:** Effect of the sampling geometry on partition‐encoding aliasing at *R* = 25. Top: *k*‐space sampling patterns for (a) purely 2D Poisson‐disk undersampling and (b) a hybrid pattern combining Poisson‐disk and equidistant undersampling along both phase‐encoding directions. Bottom: corresponding sagittal MIP views reconstructed using identical compressed sensing reconstructions. Coherent aliasing along the partition‐encoding direction (*y*‐axis) is observed only for the hybrid sampling pattern, while the Poisson‐only pattern does not exhibit such artifacts, demonstrating that partition‐direction aliasing originates from the equidistant undersampling component rather than reconstruction dimensionality.
**Table S1:** Quantitative comparison of reader study scores across reconstruction methods. Mean ± standard deviation values are shown for each qualitative assessment criterion, including overall image quality, sharpness, noise/SNR, aliasing artifacts, and regularization artifacts (over‐regularization). Compressed sensing (CS) and four frozen/trainable zero‐shot learning configurations (0/13, 0/13*, 12/1, and 12/1*) are compared. The asterisk (*) indicates that the trainable stages were initialized using pretrained weights. p‐values represent two‐sided paired Wilcoxon signed‐rank tests comparing each zero‐shot configuration against CS. Bolded p‐values indicate statistically significant differences (p< 0.05).

## Data Availability

A sample data that support the findings of this study is openly available at https://doi.org/10.5281/zenodo.16731625.

## References

[1] M. A. Barish and J. T. Ferrucci , “Magnetic Resonance Cholangiopancreatography,” New England Journal of Medicine 341, no. 4 (1999): 258–264, 10.1056/NEJM199907223410407. 10413739

[2] A. S. Fulcher , M. A. Turner , and G. W. Capps , “MR Cholangiography: Technical Advances and Clinical Applications,” Radiographics 19, no. 1 (1999): 25–44, 10.1148/radiographics.19.1.g99ja0525. 9925390

[3] E. Kaltenthaler , Y. Bravo Vergel , J. Chilcott , et al., “A Systematic Review and Economic Evaluation of Magnetic Resonance Cholangiopancreatography Compared With Diagnostic Endoscopic Retrograde Cholangiopancreatography,” Health Technology Assessment 8, no. 10 (2004): 1–89, 10.3310/hta8100. 14982656

[4] H. Irie , H. Honda , T. Tajima , et al., “Optimal MR Cholangiopancreatographic Sequence and Its Clinical Application,” Radiology 206, no. 2 (1998): 379–387, 10.1148/radiology.206.2.9457189. 9457189

[5] L. S. Yoon , O. A. Catalano , S. Fritz , C. R. Ferrone , P. F. Hahn , and D. V. Sahani , “Another Dimension in Magnetic Resonance Cholangiopancreatography: Comparison of 2‐ and 3‐Dimensional Magnetic Resonance Cholangiopancreatography for the Evaluation of Intraductal Papillary Mucinous Neoplasm of the Pancreas,” Journal of Computer Assisted Tomography 33, no. 3 (2009): 363–368, 10.1097/RCT.0b013e3181852193. 19478628

[6] T. Nakaura , M. Kidoh , N. Maruyama , et al., “Usefulness of the SPACE Pulse Sequence at 1.5T MR Cholangiography: Comparison of Image Quality and Image Acquisition Time With Conventional 3D‐TSE Sequence,” Magnetic Resonance Imaging 38, no. 5 (2013): 1014–1019, 10.1002/jmri.24033. 24105679

[7] L. Cai , B. M. Yeh , A. C. Westphalen , J. Roberts , and Z. J. Wang , “3D T2‐Weighted and Gd‐EOB‐DTPA‐Enhanced 3D T1‐Weighted MR Cholangiography for Evaluation of Biliary Anatomy in Living Liver Donors,” Abdominal Radiology 42, no. 3 (2017): 842–850, 10.1007/s00261-016-0936-z. 27714420

[8] J. H. Yoon , S. M. Lee , H. J. Kang , et al., “Clinical Feasibility of 3‐Dimensional Magnetic Resonance Cholangiopancreatography Using Compressed Sensing: Comparison of Image Quality and Diagnostic Performance,” Investigative Radiology 52, no. 10 (2017): 612–619, 10.1097/RLI.0000000000000380. 28448309

[9] S. Morita , E. Ueno , K. Suzuki , et al., “Navigator‐Triggered Prospective Acquisition Correction (PACE) Technique vs. Conventional Respiratory‐Triggered Technique for Free‐Breathing 3D MRCP: An Initial Prospective Comparative Study Using Healthy Volunteers,” Magnetic Resonance Imaging 28, no. 3 (2008): 673–677, 10.1002/jmri.21485. 18777550

[10] J. F. Glockner , M. Saranathan , E. Bayram , and C. U. Lee , “Breath‐Held MR Cholangiopancreatography (MRCP) Using a 3D Dixon Fat–Water Separated Balanced Steady State Free Precession Sequence,” Magnetic Resonance Imaging 31, no. 8 (2013): 1263–1270, 10.1016/j.mri.2013.06.008. 23876262 PMC4054824

[11] A. Almehdar and G. B. Chavhan , “MR Cholangiopancreatography at 3.0 T in Children: Diagnostic Quality and Ability in Assessment of Common Paediatric Pancreatobiliary Pathology,” British Journal of Radiology 86, no. 1025 (2013): 20130036, 10.1259/bjr.20130036. 23457194 PMC3635804

[12] K. R. Nandalur , H. K. Hussain , W. J. Weadock , et al., “Possible Biliary Disease: Diagnostic Performance of High‐Spatial‐Resolution Isotropic 3D T2‐Weighted MRCP,” Radiology 249, no. 3 (2008): 883–890, 10.1148/radiol.2493080389. 18941164

[13] M. Zins , “Breath‐Holding 3D MRCP: The Time Is Now?,” European Radiology 28, no. 9 (2018): 3719–3720, 10.1007/s00330-018-5550-8. 29931559

[14] J. G. Nam , J. M. Lee , H. J. Kang , et al., “GRASE Revisited: Breath‐Hold Three‐Dimensional (3D) Magnetic Resonance Cholangiopancreatography Using a Gradient and Spin Echo (GRASE) Technique at 3T,” European Radiology 28, no. 9 (2018): 3721–3728, 10.1007/s00330-017-5275-0. 29392471

[15] M. Yoshida , T. Nakaura , T. Inoue , et al., “Magnetic Resonance Cholangiopancreatography With GRASE Sequence at 3.0T: Does It Improve Image Quality and Acquisition Time as Compared With 3D TSE?,” European Radiology 28, no. 6 (2018): 2436–2443, 10.1007/s00330-017-5240-y. 29335869

[16] B. Sun , Z. Chen , Q. Duan , et al., “Rapid 3D Navigator‐Triggered MR Cholangiopancreatography With SPACE Sequence at 3T: Only One‐Third Acquisition Time of Conventional 3D SPACE Navigator‐Triggered MRCP,” Abdominal Radiology 45, no. 1 (2020): 134–140, 10.1007/s00261-019-02342-3. 31781898

[17] H. Chandarana , A. M. Doshi , A. Shanbhogue , et al., “Three‐Dimensional MR Cholangiopancreatography in a Breath Hold With Sparsity‐Based Reconstruction of Highly Undersampled Data,” Radiology 280, no. 2 (2016): 585–594, 10.1148/radiol.2016151935. 26982678 PMC4949145

[18] L. Zhu , X. Wu , Z. Sun , et al., “Compressed‐Sensing Accelerated 3‐Dimensional Magnetic Resonance Cholangiopancreatography: Application in Suspected Pancreatic Diseases,” Investigative Radiology 53, no. 3 (2018): 150–157, 10.1097/RLI.0000000000000421. 28976478

[19] L. Zhu , H. Xue , Z. Sun , et al., “Modified Breath‐Hold Compressed‐Sensing 3D MR Cholangiopancreatography With a Small Field‐Of‐View and High Resolution Acquisition: Clinical Feasibility in Biliary and Pancreatic Disorders,” Journal of Magnetic Resonance Imaging 48, no. 5 (2018): 1389–1399, 10.1002/jmri.26049. 29656611

[20] L. Zhu , S. Z. yong , X. H. dan , et al., “Patient‐Adapted Respiratory Training: Effect on Navigator‐Triggered 3D MRCP in Painful Pancreatobiliary Disorders,” Magnetic Resonance Imaging 45 (2018): 43–50, 10.1016/j.mri.2017.09.014. 28963048

[21] M. Lustig , D. Donoho , and J. M. Pauly , “Sparse MRI: The Application of Compressed Sensing for Rapid MR Imaging,” Magnetic Resonance in Medicine 58, no. 6 (2007): 1182–1195, 10.1002/mrm.21391. 17969013

[22] C. Plathow , S. Ley , J. Zaporozhan , et al., “Assessment of Reproducibility and Stability of Different Breath‐Hold Maneuvres by Dynamic MRI: Comparison Between Healthy Adults and Patients With Pulmonary Hypertension,” European Radiology 16, no. 1 (2006): 173–179, 10.1007/s00330-005-2795-9. 15968516

[23] M. Kocaoglu , A. S. Pednekar , H. Wang , T. Alsaied , M. D. Taylor , and M. S. Rattan , “Breath‐Hold and Free‐Breathing Quantitative Assessment of Biventricular Volume and Function Using Compressed SENSE: A Clinical Validation in Children and Young Adults,” Journal of Cardiovascular Magnetic Resonance 22, no. 1 (2020): 54, 10.1186/s12968-020-00642-y. 32713347 PMC7384228

[24] R. Otazo , D. Kim , L. Axel , and D. K. Sodickson , “Combination of Compressed Sensing and Parallel Imaging for Highly Accelerated First‐Pass Cardiac Perfusion MRI,” Magnetic Resonance in Medicine 64, no. 3 (2010): 767–776, 10.1002/mrm.22463. 20535813 PMC2932824

[25] P. Pandit , J. Rivoire , K. King , and X. Li , “Accelerated T1ρ Acquisition for Knee Cartilage Quantification Using Compressed Sensing and Data‐Driven Parallel Imaging: A Feasibility Study,” Magnetic Resonance in Medicine 75, no. 3 (2016): 1256–1261, 10.1002/mrm.25702. 25885368 PMC4609215

[26] H. K. Aggarwal , M. P. Mani , and M. Jacob , “MoDL: Model‐Based Deep Learning Architecture for Inverse Problems,” IEEE Transactions on Medical Imaging 38, no. 2 (2019): 394–405, 10.1109/TMI.2018.2865356. 30106719 PMC6760673

[27] J. Kim , M. D. Nickel , and F. Knoll , “Deep Learning‐Based Accelerated MR Cholangiopancreatography Without Fully‐Sampled Data,” NMR in Biomedicine 38, no. 3 (2025): e70002, 10.1002/nbm.70002. 39907193 PMC11795733

[28] B. Yaman , S. A. H. Hosseini , S. Moeller , J. Ellermann , K. Uğurbil , and M. Akçakaya , “Self‐Supervised Learning of Physics‐Guided Reconstruction Neural Networks Without Fully Sampled Reference Data,” Magnetic Resonance in Medicine 84, no. 6 (2020): 3172–3191, 10.1002/mrm.28378. 32614100 PMC7811359

[29] B. Yaman , S. A. H. Hosseini , and M. Akcakaya , “Zero‐Shot Self‐Supervised Learning for MRI Reconstruction,” in International Conference on Learning Representations (2022), https://openreview.net/forum?id=085y6YPaYjP.

[30] M. Akçakaya , S. Moeller , S. Weingärtner , and K. Uğurbil , “Scan‐Specific Robust Artificial‐Neural‐Networks for k‐Space Interpolation (RAKI) Reconstruction: Database‐Free Deep Learning for Fast Imaging,” Magnetic Resonance in Medicine 81, no. 1 (2019): 439–453, 10.1002/mrm.27420. 30277269 PMC6258345

[31] C. Zhang , S. Moeller , O. B. Demirel , K. Uğurbil , and M. Akçakaya , “Residual RAKI: A Hybrid Linear and Non‐Linear Approach for Scan‐Specific k‐Space Deep Learning,” NeuroImage 256 (2022): 119248, 10.1016/j.neuroimage.2022.119248. 35487456 PMC9179026

[32] V. Lempitsky , A. Vedaldi , and D. Ulyanov , “Deep Image Prior,” in 2018 IEEE/CVF Conference on Computer Vision and Pattern Recognition (IEEE, 2018), 9446–9454, 10.1109/CVPR.2018.00984.

[33] L. Shen , J. Pauly , and L. Xing , “NeRP: Implicit Neural Representation Learning With Prior Embedding for Sparsely Sampled Image Reconstruction,” IEEE Transactions on Neural Networks and Learning Systems 35, no. 1 (2024): 770–782, 10.1109/TNNLS.2022.3177134. PMC10889906 35657845

[34] R. Feng , Q. Wu , J. Feng , et al., “IMJENSE: Scan‐Specific Implicit Representation for Joint Coil Sensitivity and Image Estimation in Parallel MRI,” IEEE Transactions on Medical Imaging 43, no. 4 (2024): 1539–1553, 10.1109/TMI.2023.3342156. 38090839

[35] J. Cho , Y. Jun , X. Wang , C. Kobayashi , and B. Bilgic , “Improved Multi‐Shot Diffusion‐Weighted MRI With Zero‐Shot Self‐Supervised Learning Reconstruction,” in Medical Image Computing and Computer Assisted Intervention – MICCAI 2023, vol. 14220, Lecture Notes in Computer Science, ed. H. Greenspan , A. Madabhushi , P. Mousavi , et al. (Springer Nature, 2023), 457–466, 10.1007/978-3-031-43907-0_44.

[36] M. P. Lichy , B. M. Wietek , J. P. Mugler , et al., “Magnetic Resonance Imaging of the Body Trunk Using a Single‐Slab, 3‐Dimensional, T2‐Weighted Turbo‐Spin‐Echo Sequence With High Sampling Efficiency (SPACE) for High Spatial Resolution Imaging: Initial Clinical Experiences,” Investigative Radiology 40, no. 12 (2005): 754–760, 10.1097/01.rli.0000185880.92346.9e. 16304477

[37] S. J. Inati , J. D. Naegele , N. R. Zwart , et al., “ISMRM Raw Data Format: A Proposed Standard for MRI Raw Datasets,” Magnetic Resonance in Medicine 77, no. 1 (2017): 411–421, 10.1002/mrm.26089. 26822475 PMC4967038

[38] W. T. Clarke , “Python port of mapVBVD,” accessed July 2, 2025, https://github.com/wtclarke/pymapvbvd.

[39] M. Uecker , P. Lai , M. J. Murphy , et al., “ESPIRiT—An Eigenvalue Approach to Autocalibrating Parallel MRI: Where SENSE Meets GRAPPA,” Magnetic Resonance in Medicine 71, no. 3 (2014): 990–1001, 10.1002/mrm.24751. 23649942 PMC4142121

[40] F. Ong and M. Lustig , “SigPy: A Python Package for High Performance Iterative Reconstruction,” Proceedings of the International Society for Magnetic Resonance in Medicine 27 (2019): 4819, https://cds.ismrm.org/protected/19MProceedings/PDFfiles/4819.html.

[41] K. He , X. Zhang , S. Ren , and J. Sun , “Deep Residual Learning for Image Recognition,” in 2016 IEEE Conference on Computer Vision and Pattern Recognition (CVPR) (IEEE, 2016), 770–778, 10.1109/CVPR.2016.90.

[42] I. Loshchilov and F. Hutter , “SGDR: Stochastic Gradient Descent With Warm Restarts,” in ICLR 2017 (2017), 10.48550/arXiv.1608.03983.

[43] M. A. Griswold , P. M. Jakob , R. M. Heidemann , et al., “Generalized Autocalibrating Partially Parallel Acquisitions (GRAPPA),” Magnetic Resonance in Medicine 47, no. 6 (2002): 1202–1210, 10.1002/mrm.10171. 12111967

[44] N. McKibben , “Python Implementations of GRAPPA‐Like Algorithms,” accessed July 3, 2025, https://github.com/mckib2/pygrappa.

[45] J. Wang , D. An , and J. P. Haldar , “The ‘Hidden Noise’ Problem in MR Image Reconstruction,” Magnetic Resonance in Medicine 92, no. 3 (2024): 982–996, 10.1002/mrm.30100. 38576156 PMC11209803

[46] Z. Deng , B. Yaman , C. Zhang , S. Moeller , and M. Akçakaya , “Efficient Training of 3D Unrolled Neural Networks for MRI Reconstruction Using Small Databases,” in 2021 55th Asilomar Conference on Signals, Systems, and Computers (IEEE, 2021), 886–889, 10.1109/IEEECONF53345.2021.9723247.

[47] A. Avesta , S. Hossain , M. Lin , M. Aboian , H. M. Krumholz , and S. Aneja , “Comparing 3D, 2.5D, and 2D Approaches to Brain Image Auto‐Segmentation,” Bioengineering 10, no. 2 (2023): 181, 10.3390/bioengineering10020181. 36829675 PMC9952534

[48] K. Wang , M. Kellman , C. M. Sandino , et al., “Memory‐Efficient Learning for High‐Dimensional MRI Reconstruction,” in Medical Image Computing and Computer Assisted Intervention – MICCAI 2021, vol. 12906. Lecture Notes in Computer Science., ed. M. De Bruijne , P. C. Cattin , S. Cotin , et al. (Springer International Publishing, 2021), 461–470, 10.1007/978-3-030-87231-1_45.

[49] F. Gadjimuradov , T. Benkert , M. D. Nickel , and A. Maier , “Robust Partial Fourier Reconstruction for Diffusion‐Weighted Imaging Using a Recurrent Convolutional Neural Network,” Magnetic Resonance in Medicine 87, no. 4 (2022): 2018–2033, 10.1002/mrm.29100. 34841550

