# Supplementary material for: Zero‐Shot Self‐Supervised Learning of Single Breath‐Hold Magnetic Resonance Cholangiopancreatography (MRCP) Reconstruction
Source: Magn Reson Med. 2026 Jun 12;96(4):1666–81. doi: 10.1002/mrm.70467 (PMC13419269; doi:10.1002/mrm.70467)
Supplement: Supplementary file 1 — Figure S1: Undersampling pattern for breath‐hold MRCP, combining 2D Poisson‐disk incoherent undersampling with partial Fourier undersampling, leading to a total acceleration factor of R = 25 and 14‐s breath‐hold scans. The x‐ and y‐axis correspond to the in‐plane phase encoding and partition encoding directions, respectively. The fully sampled readout (frequency encoding) direction is orthogonal to the phase‐partition encoding plane (i.e., through‐plane). Figure S2: Full respiratory navigator signals corresponding to Figure 3d,e are shown in (a) and (b), respectively. Acquisition time was 360 s for (a) and 587 s for (b). Red boxes indicate the regions shown in the zoomed‐in views in Figure 3. The x‐axis represents the signal sampling index, and the y‐axis shows acquired projections of the liver‐dome navigator. Figure S3: Reconstruction results from a 33‐year‐old male (volunteer #01). Reconstruction methods include a triggered acquisition (Triggered) and a 14 s breath‐hold acquisition reconstructed with compressed sensing (CS), a pretrained reconstruction model used for the frozen stages of ZS 12/1* (Pretrained), zero‐shot learning with frozen/trainable configurations of 0/13 and 12/1, respectively. The asterisk (*) indicates that the network for the trainable stages is initialized with the pretrained weights. For each method, three visualizations are provided: a coronal maximum intensity projection (MIP, top left), a cropped coronal MIP focused on the region of interest (bottom left), and a sagittal MIP (right). The red box in the coronal MIP marks the ROI shown in the cropped view. Figure S4: Reconstruction results from a 27‐year‐old female (volunteer #03). Reconstruction methods include a triggered acquisition (Triggered) and a 14 s breath‐hold acquisition reconstructed with compressed sensing (CS), a pretrained reconstruction model used for the frozen stages of ZS 12/1* (Pretrained), zero‐shot learning with frozen/trainable configurations of 0/13 and 12/1, respec [file MRM-96-1666-s001.docx]

Zero-shot self-supervised learning of single breath-hold magnetic resonance cholangiopancreatography (MRCP) reconstruction

Jinho Kim^1,2^, Marcel Dominik Nickel^2^, Florian Knoll^1^

^1^Department Artificial Intelligence in Biomedical Engineering, Friedrich-Alexander-Universität Erlangen-Nürnberg, Erlangen, Germany

^2^Research and Clinical Translation, Magnetic Resonance, Siemens Healthineers AG, Erlangen, Germany

| Correspondence to: | Jinho Kim Computational Imaging Lab Department Artificial Intelligence in Biomedical Engineering Friedrich-Alexander-Universität Erlangen-Nürnberg Nürnberger Straße 74, D-91054 Erlangen, Germany E-mail: jinho.kim@fau.de |
| --- | --- |

# Supporting Information

## A - Undersampling pattern for breath-hold MRCP

| 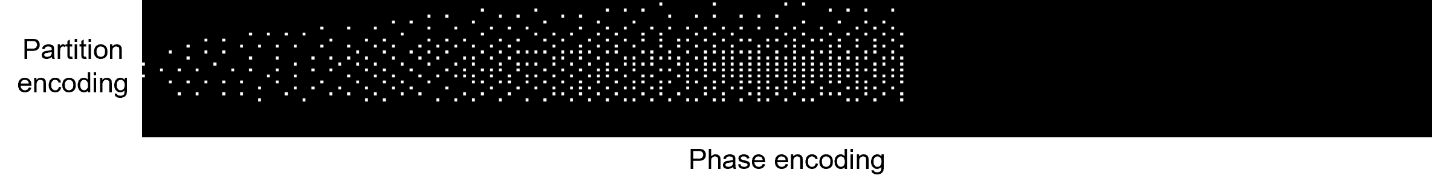  Figure S1 Undersampling pattern for breath-hold MRCP, combining 2D Poisson-disk incoherent undersampling with partial Fourier undersampling, leading to a total acceleration factor of R=25 and 14-second breath-hold scans. The x- and y-axis correspond to the in-plane phase encoding and partition encoding directions, respectively. The fully sampled readout (frequency encoding) direction is orthogonal to the phase-partition encoding plane (i.e., through-plane). |
| --- |

## B - Deep learning-based scan-specific reconstruction

Additional deep learning-based scan-specific reconstructions, such as deep image prior^1^ (DIP) and implicit neural representation^2,3^ (INR) were compared with zero-shot self-supervised reconstruction^4^ for 3D breath-hold MRCP. The forward model of MRI reconstruction is defined as follow,

|  | $y=Ax\mathcal{+E}$, | ( 1 ) |
| --- | --- | --- |

where $y\in\mathbb{C}^{M}$ denotes multi-coil undersampled k-space data, $x\in\mathbb{C}^{N}$ the $N$-pixel reconstructed image, $A\in\mathbb{C}^{M\times N}$ the encoding operator containing undersampling, Fourier transform, and coil sensitivities, and $\mathcal{E\in}\mathbb{C}^{M}$ measurement noise.

DIP reconstruction was implemented based on a ResNet architecture consisting of 20 residual blocks with 128 feature channels. The trainable code vector $z\in\mathbb{R}^{N\times2}$ was initialized with independent and identically distributed (i.i.d.) samples from the uniform distribution for real and imaginary values at each channel. In this formulation, the trainable code vector $z$ has the same spatial dimensionality as the reconstructed image and thus acts as an image-domain input rather than a low-dimensional latent code. During optimization, $z$ was additionally regularized by additive noise^1^, $\tilde{z}\leftarrow z+\alpha\boldsymbol{\epsilon}$, where $\boldsymbol{\epsilon\in}\mathbb{R}^{N\times2}$ is element-wise sampled noise from the i.i.d. Gaussian distribution, and $\alpha$ is a trainable noise scaling parameter initialized with 0.01. The DIP model was optimized for 200 iterations per each readout sample. The number of iterations was selected based on empirical convergence analysis, as larger iteration numbers led to overfitting and degradation of image quality. The objective function combining a normalized $\mathcal{l}_{1}$-$\mathcal{l}_{2}$ loss in the k-space domain and a total variation (TV) loss on the image magnitude takes the form,

|  | $\underset{\theta,z,\lambda_{TV},\alpha}{\mathrm{argmin}} \mathcal{L}_{\mathcal{l}_{1}\text{-}\mathcal{l}_{2}}\left( A\tilde{x},y \right)+\lambda_{TV}\mathcal{L}_{TV}\left( \left\vert\tilde{x} \right\vert\right)$, | ( 2 ) |
| --- | --- | --- |

with

|  | $\tilde{x}=\mathcal{N}_{\theta}^{DIP}\left( \tilde{z} \right),$ |  |
| --- | --- | --- |

where $\tilde{x}\in\mathbb{C}^{N}$ denotes an implicitly regularized image, $\mathcal{N}_{\theta}^{DIP}: \mathbb{R}^{N\times2}\to\mathbb{C}^{N}$ a neural network for DIP reconstruction parameterized with $\theta$, $\lambda_{TV}$ a trainable balancing parameter initialized with 10. Optimization was performed using the Adam optimizer with a learning rate of $1\times{10}^{-4}$, scheduled using cosine annealing.

INR reconstruction was adapted from scan-specific implicit representation for joint coil sensitivity and image estimation in parallel MRI^3^ (IMJENSE). In contrast to the original IMJENSE, the pre-estimated coil sensitivity maps were used to keep consistency over reconstructions. Hash encoding was utilized to speed up the reconstruction with following encoding configurations: 4 multi-resolution levels, 2 features per level, a hash table size of $2^{18}$, a base resolution of 5, and a per-level scale factor of 2. The INR model was optimized for 1000 iterations per each readout sample. The objective function combining a $\mathcal{l}_{1}$ loss in the k-space domain and a total variation (TV) loss on the magnitude image takes the form,

|  | $\underset{\theta}{\mathrm{argmin}} \mathcal{L}_{\mathcal{l}_{1}}\left( A\tilde{x} ,y \right)+\lambda_{TV}\mathcal{L}_{TV}\left( \left\vert\tilde{x} \right\vert\right)$, | ( 3 ) |
| --- | --- | --- |

with

|  | $\tilde{x}=\mathcal{N}_{\theta}^{INR}\left( i \right),$ |  |
| --- | --- | --- |

where $\tilde{x}\in\mathbb{C}^{N}$ denotes a discretized image vector, $\mathcal{N}_{\theta}: \mathbb{R}^{N\times2}\to\mathbb{C}^{N}$ a neural network for INR reconstruction parameterized with $\theta$, $i\in\mathbb{R}^{N\times2}$ the normalized 2D spatial coordinates corresponding to the discretized image grid. The fixed parameter, $\lambda_{TV}$, was initialized with 0.4 to balance the relative contributions of the two loss terms, which have substantially different value scales. Optimization was performed using the Adam optimizer with a learning rate of $1\times{10}^{-2}$, scheduled using cosine annealing.

Hyperparameters for DIP and INR were selected through extensive empirical tuning to maximize reconstruction quality under a fixed computational budget and iteration schedule. While we cannot exclude the possibility that alternative configurations may further improve their performance, the chosen settings provided stable convergence and visually optimal reconstructions across all evaluated subjects.

## C - Full respiratory traces of respiratory-triggered MRCP acquisition

| 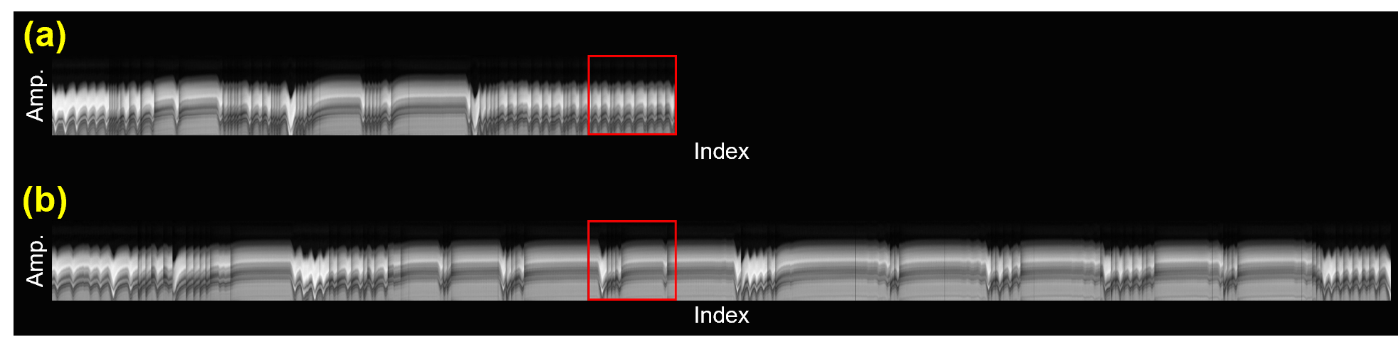  Figure S2 Full respiratory navigator signals corresponding to Figures 3d and 3e are shown in (a) and (b), respectively. Acquisition time was 360s for (a) and 587s for (b). Red boxes indicate the regions shown in the zoomed-in views in Figure 3. The x-axis represents the signal sampling index, and the y-axis shows acquired projections of the liver-dome navigator. |
| --- |

## D - Zero-shot reconstruction results of additional subjects

| 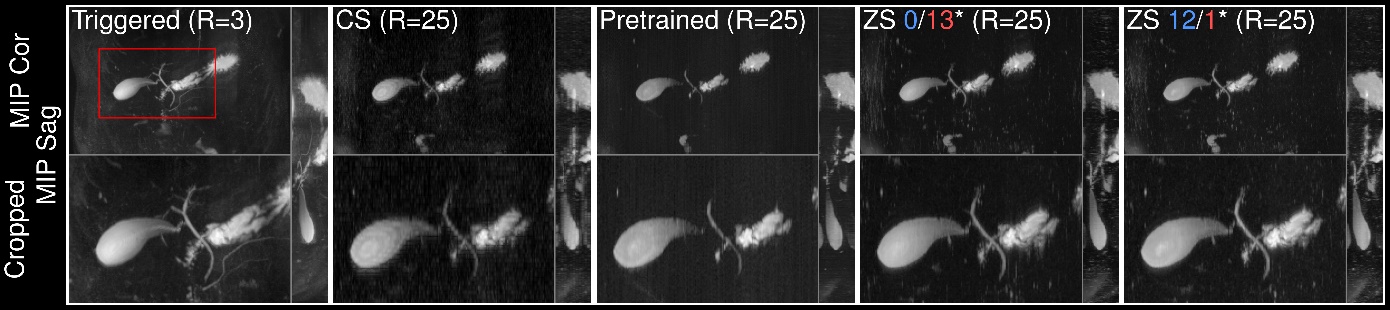  Figure S3 Reconstruction results from a 33-year-old male (volunteer #01). Reconstruction methods include a triggered acquisition (Triggered) and a 14s breath-hold acquisition reconstructed with compressed sensing (CS), a pretrained reconstruction model used for the frozen stages of ZS 12/1* (Pretrained), zero-shot learning with frozen/trainable configurations of 0/13 and 12/1, respectively. The asterisk (*) indicates that the network for the trainable stages is initialized with the pretrained weights. For each method, three visualizations are provided: a coronal maximum intensity projection (MIP, top left), a cropped coronal MIP focused on the region of interest (bottom left), and a sagittal MIP (right). The red box in the coronal MIP marks the ROI shown in the cropped view. |
| --- |

| 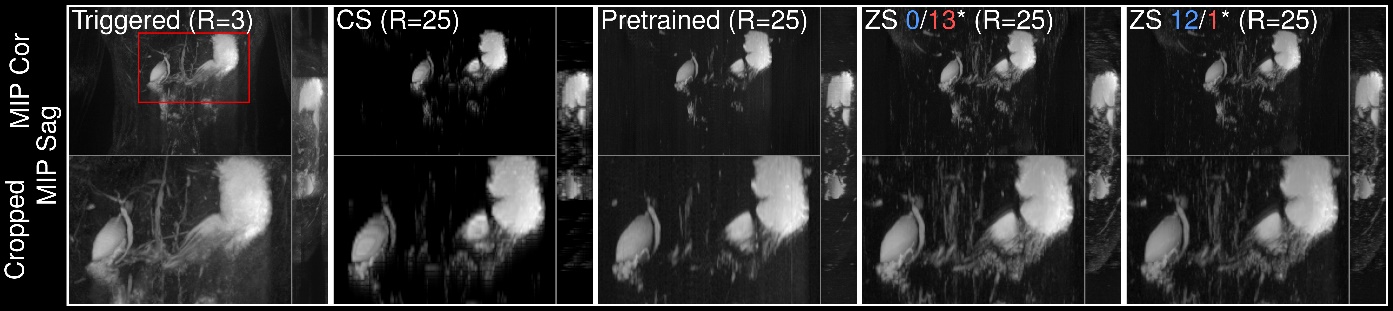  Figure S4 Reconstruction results from a 27-year-old female (volunteer #03). Reconstruction methods include a triggered acquisition (Triggered) and a 14s breath-hold acquisition reconstructed with compressed sensing (CS), a pretrained reconstruction model used for the frozen stages of ZS 12/1* (Pretrained), zero-shot learning with frozen/trainable configurations of 0/13 and 12/1, respectively. The asterisk (*) indicates that the network for the trainable stages is initialized with the pretrained weights. For each method, three visualizations are provided: a coronal maximum intensity projection (MIP, top left), a cropped coronal MIP focused on the region of interest (bottom left), and a sagittal MIP (right). The red box in the coronal MIP marks the ROI shown in the cropped view. |  |
| --- | --- |
| 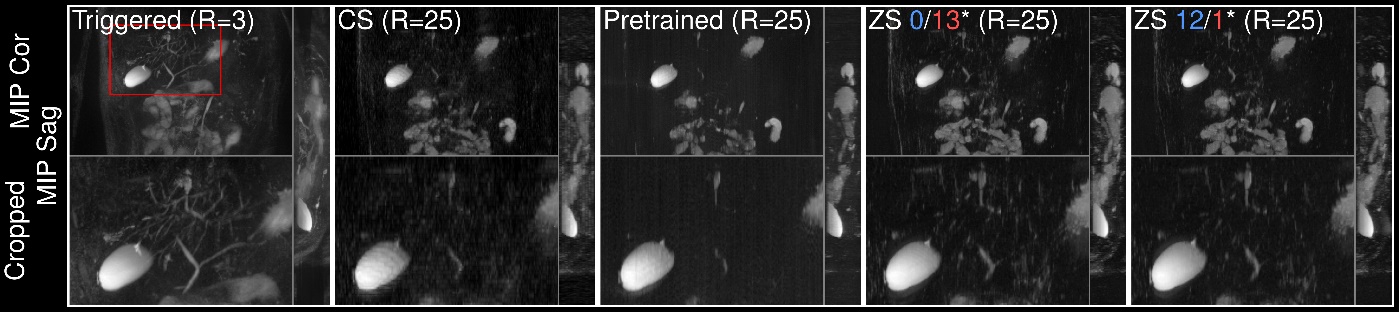  Figure S5 Reconstruction results from a 27-year-old male (volunteer #04). Reconstruction methods include a triggered acquisition (Triggered) and a 14s breath-hold acquisition reconstructed with compressed sensing (CS), a pretrained reconstruction model used for the frozen stages of ZS 12/1* (Pretrained), zero-shot learning with frozen/trainable configurations of 0/13 and 12/1, respectively. The asterisk (*) indicates that the network for the trainable stages is initialized with the pretrained weights. For each method, three visualizations are provided: a coronal maximum intensity projection (MIP, top left), a cropped coronal MIP focused on the region of interest (bottom left), and a sagittal MIP (right). The red box in the coronal MIP marks the ROI shown in the cropped view. | |
| 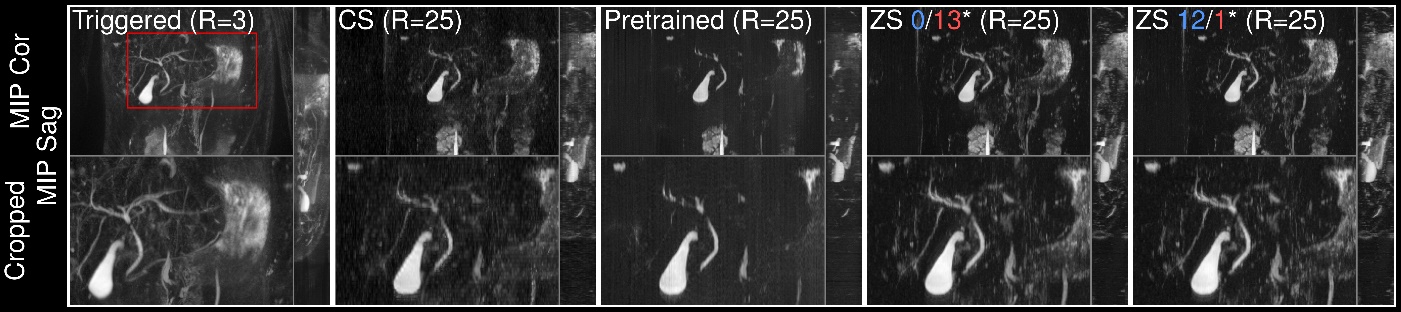  Figure S6 Reconstruction results from a 29-year-old male (volunteer #05). Reconstruction methods include a triggered acquisition (Triggered) and a 14s breath-hold acquisition reconstructed with compressed sensing (CS), a pretrained reconstruction model used for the frozen stages of ZS 12/1* (Pretrained), zero-shot learning with frozen/trainable configurations of 0/13 and 12/1, respectively. The asterisk (*) indicates that the network for the trainable stages is initialized with the pretrained weights. For each method, three visualizations are provided: a coronal maximum intensity projection (MIP, top left), a cropped coronal MIP focused on the region of interest (bottom left), and a sagittal MIP (right). The red box in the coronal MIP marks the ROI shown in the cropped view. | |
| 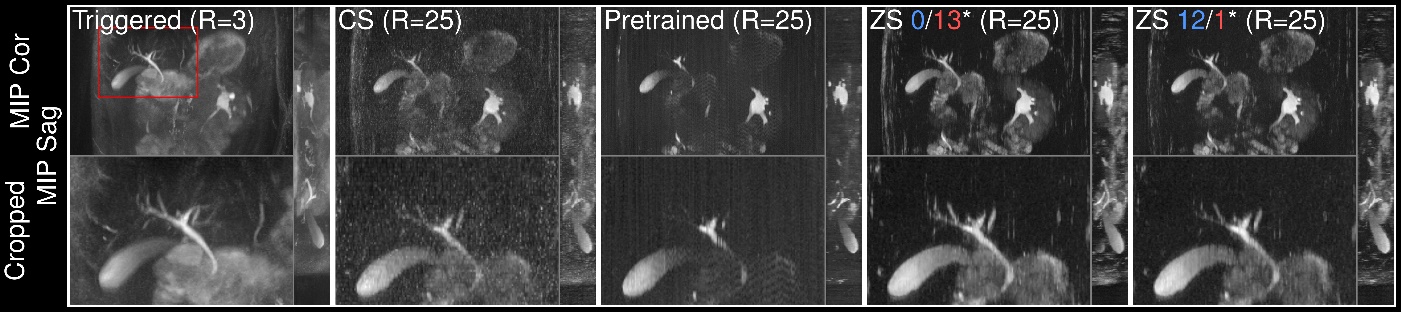  Figure S7 Reconstruction results from a 28-year-old male (volunteer #06). Reconstruction methods include a triggered acquisition (Triggered) and a 14s breath-hold acquisition reconstructed with compressed sensing (CS), a pretrained reconstruction model used for the frozen stages of ZS 12/1* (Pretrained), zero-shot learning with frozen/trainable configurations of 0/13 and 12/1, respectively. The asterisk (*) indicates that the network for the trainable stages is initialized with the pretrained weights. For each method, three visualizations are provided: a coronal maximum intensity projection (MIP, top left), a cropped coronal MIP focused on the region of interest (bottom left), and a sagittal MIP (right). The red box in the coronal MIP marks the ROI shown in the cropped view. | |
| 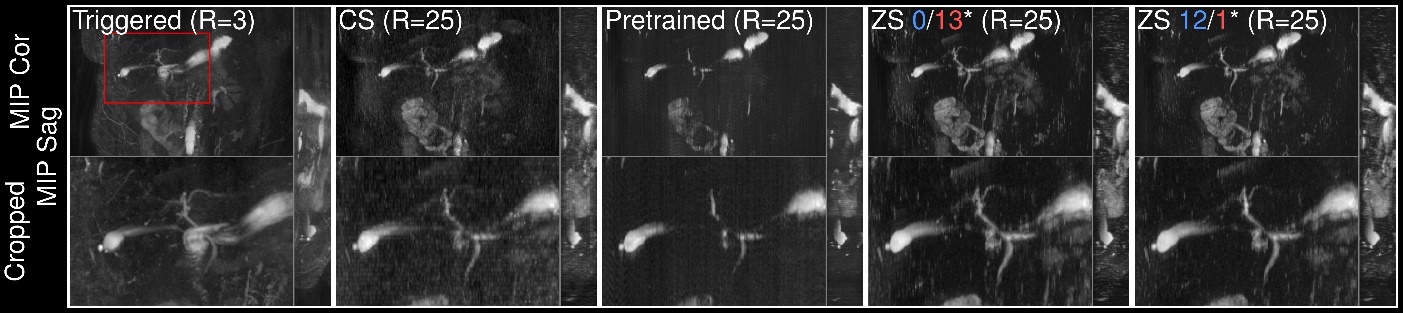  Figure S8 Reconstruction results from a 46-year-old female (volunteer #09). Reconstruction methods include a triggered acquisition (Triggered) and a 14s breath-hold acquisition reconstructed with compressed sensing (CS), a pretrained reconstruction model used for the frozen stages of ZS 12/1* (Pretrained), zero-shot learning with frozen/trainable configurations of 0/13 and 12/1, respectively. The asterisk (*) indicates that the network for the trainable stages is initialized with the pretrained weights. For each method, three visualizations are provided: a coronal maximum intensity projection (MIP, top left), a cropped coronal MIP focused on the region of interest (bottom left), and a sagittal MIP (right). The red box in the coronal MIP marks the ROI shown in the cropped view. | |
| 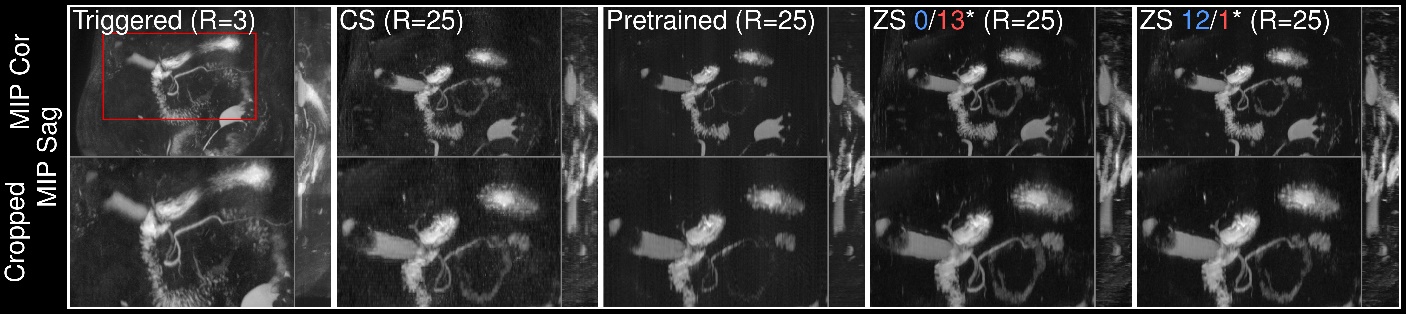  Figure S9 Reconstruction results from a 68-year-old male (volunteer #10). Reconstruction methods include a triggered acquisition (Triggered) and a 14s breath-hold acquisition reconstructed with compressed sensing (CS), a pretrained reconstruction model used for the frozen stages of ZS 12/1* (Pretrained), zero-shot learning with frozen/trainable configurations of 0/13 and 12/1, respectively. The asterisk (*) indicates that the network for the trainable stages is initialized with the pretrained weights. For each method, three visualizations are provided: a coronal maximum intensity projection (MIP, top left), a cropped coronal MIP focused on the region of interest (bottom left), and a sagittal MIP (right). The red box in the coronal MIP marks the ROI shown in the cropped view. | |
| 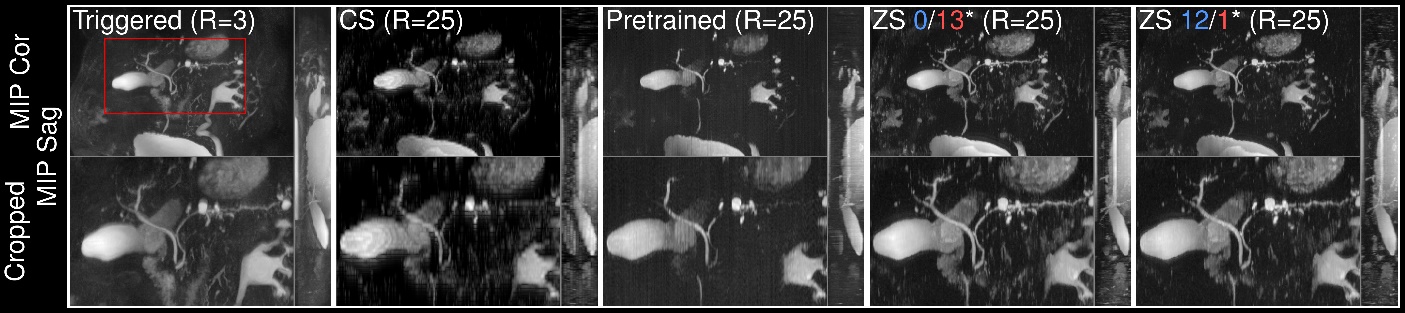  Figure S10 Reconstruction results from an 86-year-old male (volunteer #11). Reconstruction methods include a triggered acquisition (Triggered) and a 14s breath-hold acquisition reconstructed with compressed sensing (CS), a pretrained reconstruction model used for the frozen stages of ZS 12/1* (Pretrained), zero-shot learning with frozen/trainable configurations of 0/13 and 12/1, respectively. The asterisk (*) indicates that the network for the trainable stages is initialized with the pretrained weights. For each method, three visualizations are provided: a coronal maximum intensity projection (MIP, top left), a cropped coronal MIP focused on the region of interest (bottom left), and a sagittal MIP (right). The red box in the coronal MIP marks the ROI shown in the cropped view. | |

## E – Reader study

Table S1 Quantitative comparison of reader study scores across reconstruction methods. Mean ± standard deviation values are shown for each qualitative assessment criterion, including overall image quality, sharpness, noise/SNR, aliasing artifacts, and regularization artifacts (over-regularization). Compressed sensing (CS) and four frozen/trainable zero-shot learning configurations (0/13, 0/13*, 12/1, and 12/1*) are compared. The asterisk (*) indicates that the trainable stages were initialized using pretrained weights. $p$-values represent two-sided paired Wilcoxon signed-rank tests comparing each zero-shot configuration against CS. Bolded $p$-values indicate statistically significant differences ($p$<0.05).

| Field | Score | CS | 0/13 | 0/13* | 12/1 | 12/1* |
| --- | --- | --- | --- | --- | --- | --- |
| Overall image quality | Avg. | $1.73\pm0.39$ | $2.65\pm0.38$ | $2.61\pm0.33$ | $2.42\pm0.52$ | $2.56\pm0.42$ |
|  | $p$ |  | $\boldsymbol{<0.001}$ | $\boldsymbol{<0.001}$ | $\boldsymbol{0.00}\boldsymbol{2}$ | $\boldsymbol{<0.001}$ |
| Sharpness | Avg. | $2.42\pm0.42$ | $2.65\pm0.41$ | $2.73\pm0.44$ | $2.70\pm0.50$ | $2.52\pm0.52$ |
|  | $p$ |  | $0.219$ | $0.094$ | $0.219$ | $0.562$ |
| Noise/SNR | Avg. | $2.33\pm0.47$ | $3.79\pm0.31$ | $3.45\pm0.38$ | $3.58\pm0.47$ | $3.52\pm0.40$ |
|  | $p$ |  | $\boldsymbol{<0.001}$ | $\boldsymbol{0.00}\boldsymbol{2}$ | $\boldsymbol{<}\boldsymbol{0.00}\boldsymbol{1}$ | $\boldsymbol{<0.001}$ |
| Aliasing artifacts | Avg. | $1.48\pm0.43$ | $3.21\pm0.43$ | $3.27\pm0.42$ | $2.67\pm0.54$ | $2.79\pm0.45$ |
|  | $p$ |  | $\boldsymbol{<0.001}$ | $\boldsymbol{<0.001}$ | $\boldsymbol{<0.00}\boldsymbol{1}$ | $\boldsymbol{<0.001}$ |
| Regularization artifacts  (over-regularization) | Avg. | $2.67\pm0.56$ | $2.42\pm0.45$ | $2.39\pm0.53$ | $2.30\pm0.31$ | $2.42\pm0.40$ |
|  | $p$ |  | $0.250$ | $0.250$ | $0.105$ | $0.260$ |
| **Note**:   - The asterisk (*) indicates that the network for the $m$-trainable stages is initialized with the $m$-stage pretrained network. - The notation format for Average is $\text{Mean±Standard}\text{ deviation}$. - $p$-values ($p< 0.05$) denote statistically significant differences in image quality scores compared to CS. Bolded $p$-values indicate statistically significant differences.   **Abbreviations**: CS, compressed sensing; Avg, average score | | | | | | |

## F - Lightweight reconstruction strategies for zero-shot learning

| 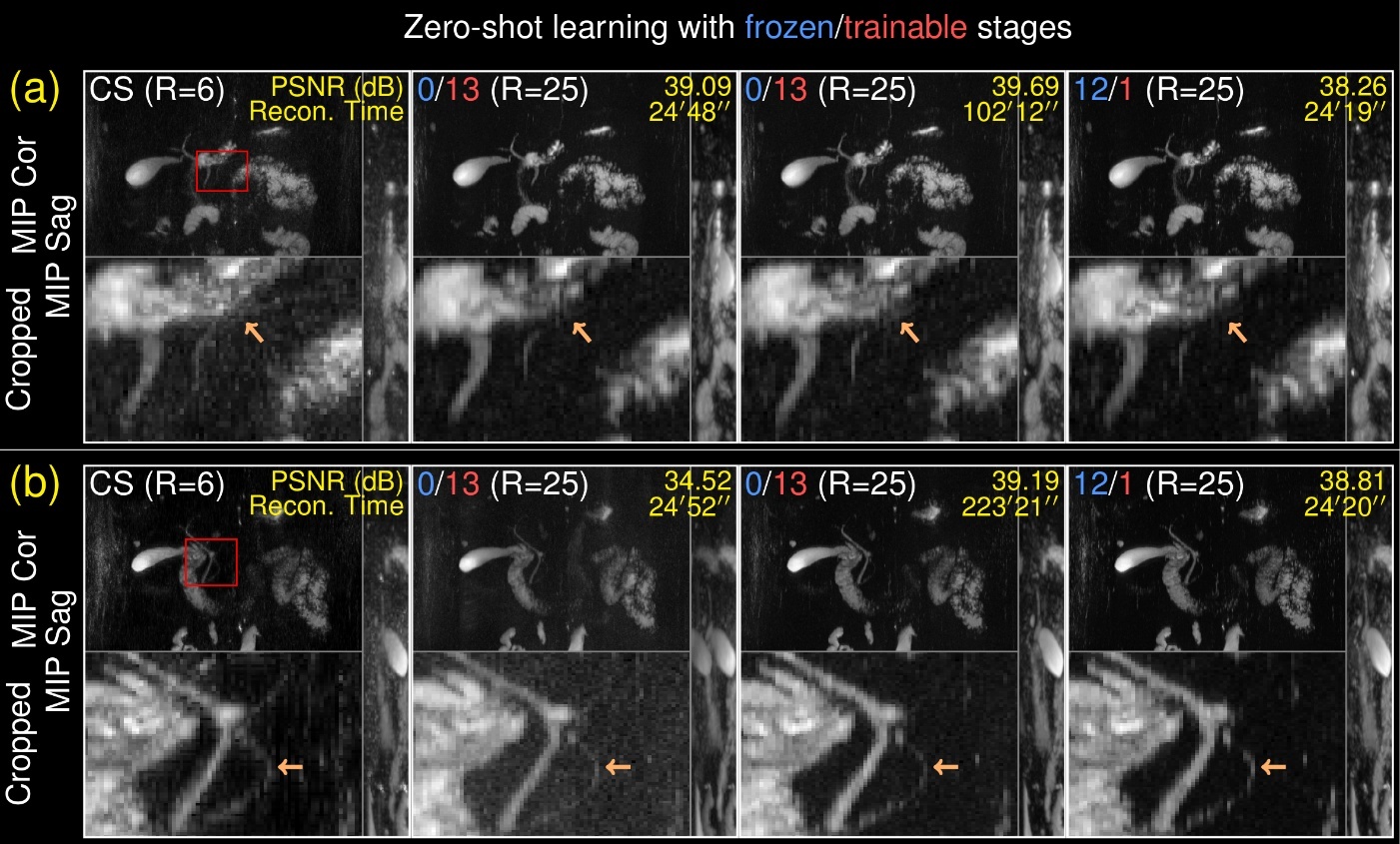  Figure S11 Reconstruction results of retrospectively undersampled acquisition from (a) a 33-year-old male (volunteer #01) and (b) a 46-year-old male (volunteer #02) using zero-shot learning. Each block shows a breath-hold acquisition at R=6 and its retrospectively undersampling at R=25, reconstructed using different frozen/trainable configurations: **middle left**, 0/13 with truncated reconstruction time; **middle right**, fully converged 0/13; and **rightmost**, 12/1. For each method, three visualizations are provided: a coronal maximum intensity projection (MIP, top left), a cropped coronal MIP focused on the region of interest (bottom left), and a sagittal MIP (right). The red box in the coronal MIP marks the ROI shown in the cropped view. Peak signal-to-noise ratio (PSNR) was computed against the original data at R=6. “Recon. Time” indicates the reconstruction time. The orange arrows highlight regions with notable differences in ductal visibility across reconstructions. |
| --- |
|  |

## G - Discontinuity artifacts along the decoupled readout direction

To support the interpretation of readout direction discontinuities, we performed a direct comparison between a joint 3D reconstruction and the readout-decoupled 2D reconstruction using identical data and sampling. The coil sensitivity maps for 3D reconstruction are estimated from decoupled 2D data and stacked into a 3D volume with spatial regularization along the readout direction. Figure S12 shows that the discontinuity artifacts were absent in the 3D reconstruction but clearly present in the decoupled 2D reconstruction, particularly in background regions, supporting their attribution to spatial correlation by the employed regularization.

| 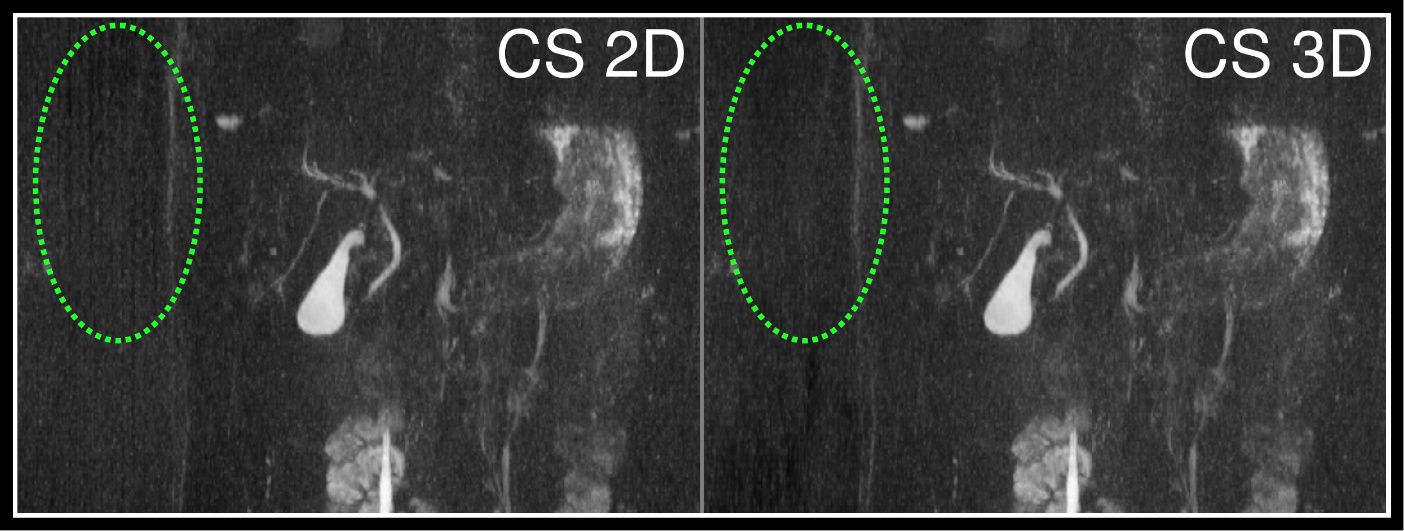  Figure S12 Comparison of readout-direction continuity between 2D and 3D reconstruction strategies. CS 2D denotes a slice-wise compressed sensing reconstruction and CS 3D denotes a joint 3D compressed sensing reconstruction. For CS 3D, coil sensitivity maps are estimated from decoupled 2D data and stacked into a 3D volume using spatial regularization along the readout direction, whereas CS 2D uses independent 2D coil sensitivity maps per readout index. |
| --- |

## H - Aliasing artifacts along the slice encoding direction

To analyze slice-encoding aliasing shown in Figure S13, we investigated the effect of sampling geometry by comparing R=25 Poisson-disk sampling patterns with and without an equidistant undersampling component along the partition-encoding direction. Figure S14 demonstrates that the pure Poisson-disk sampling did not produce aliasing artifacts, whereas the hybrid sampling pattern combining Poisson-disk and equidistant undersampling resulted in pronounced partition-direction aliasing. These controlled comparisons provide direct evidence linking the observed artifacts to reconstruction dimensionality and sampling geometry.

| 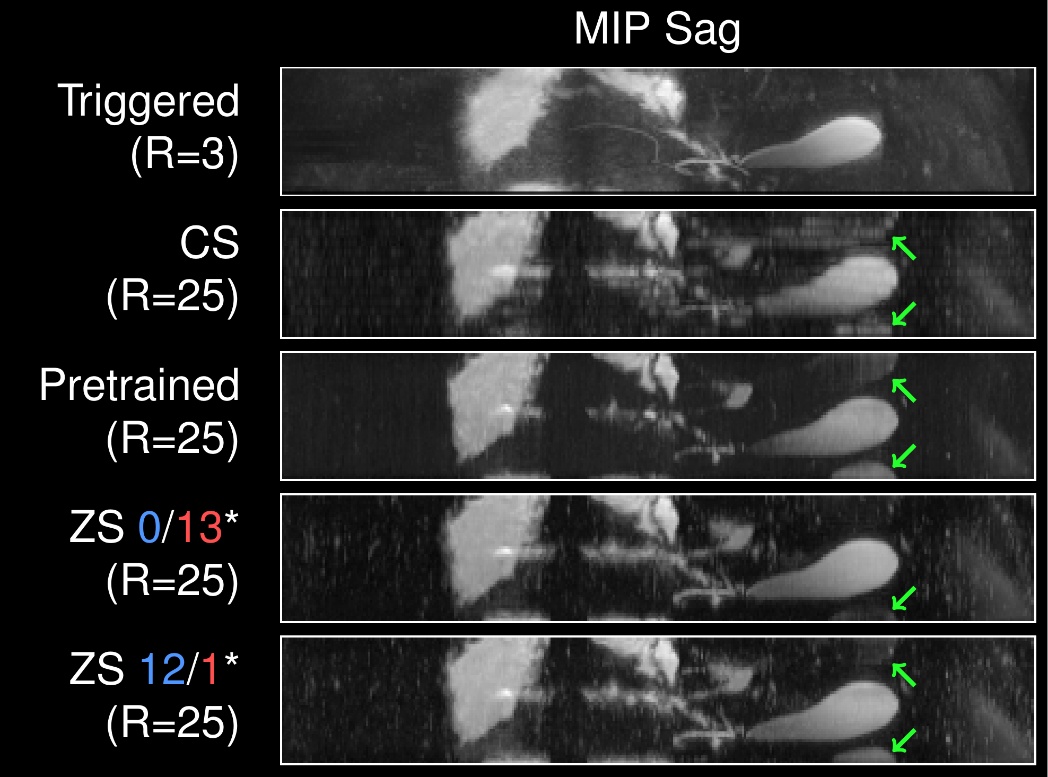  Figure S13 Reconstruction results from a 33-year-old male (volunteer #01), demonstrating aliasing artifacts along the slice encoding direction in the sagittal MIP view. Each reconstruction block shows results from a triggered acquisition (Triggered) and a 14s breath-hold acquisition reconstructed with compressed sensing (CS), a pretrained reconstruction model used for the frozen stages of ZS 12/1* (Pretrained), zero-shot learning with frozen/trainable configurations of 0/13* and 12/1*, respectively. The asterisk (*) indicates that the network for the trainable stages is initialized with the pretrained weights. Green arrows indicate areas where aliasing artifacts are observed. |
| --- |

| 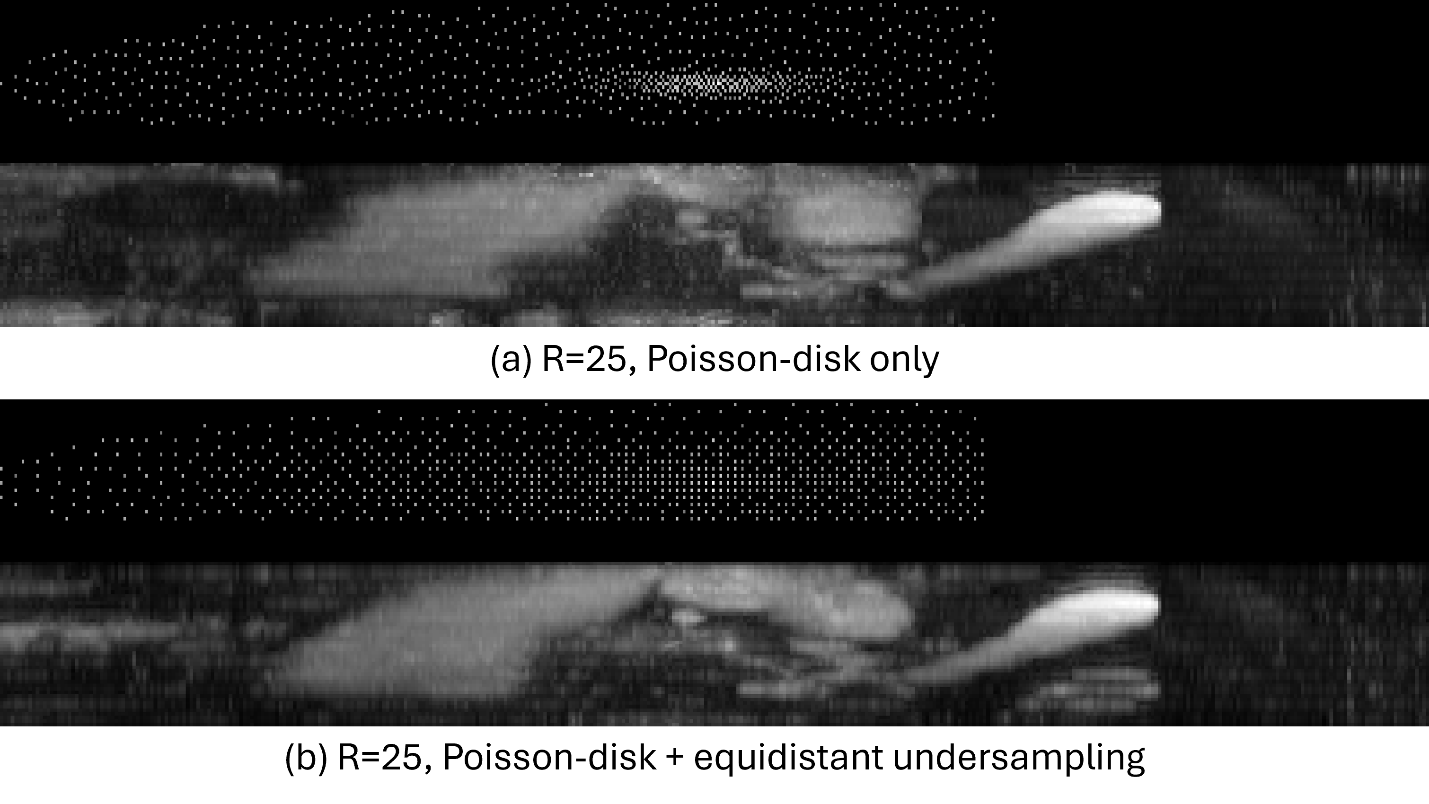  Figure S14 Effect of the sampling geometry on partition-encoding aliasing at R=25. Top: k-space sampling patterns for (a) purely 2D Poisson-disk undersampling and (b) a hybrid pattern combining Poisson-disk and equidistant undersampling along both phase-encoding directions. Bottom: corresponding sagittal MIP views reconstructed using identical compressed sensing reconstructions. Coherent aliasing along the partition-encoding direction (y-axis) is observed only for the hybrid sampling pattern, while the Poisson-only pattern does not exhibit such artifacts, demonstrating that partition-direction aliasing originates from the equidistant undersampling component rather than reconstruction dimensionality. |
| --- |

# References

1. Ulyanov D, Vedaldi A, Lempitsky V. Deep Image Prior. In: *2018 IEEE/CVF Conference on Computer Vision and Pattern Recognition*. 2018:9446-9454. doi:10.1109/CVPR.2018.00984

2. Shen L, Pauly J, Xing L. NeRP: Implicit Neural Representation Learning With Prior Embedding for Sparsely Sampled Image Reconstruction. *IEEE Trans Neural Netw Learning Syst*. 2024;35(1):770-782. doi:10.1109/TNNLS.2022.3177134

3. Feng R, Wu Q, Feng J, et al. IMJENSE: Scan-Specific Implicit Representation for Joint Coil Sensitivity and Image Estimation in Parallel MRI. *IEEE Transactions on Medical Imaging*. 2024;43(4):1539-1553. doi:10.1109/TMI.2023.3342156

4. Yaman B, Hosseini SAH, Akcakaya M. Zero-Shot Self-Supervised Learning for MRI Reconstruction. In: *International Conference on Learning Representations*. 2022. https://openreview.net/forum?id=085y6YPaYjP
